# Genome sequencing of SARS-CoV-2 omicron variants in Delhi reveals alterations in immunogenic regions in spike glycoprotein

**DOI:** 10.3389/fimmu.2023.1209513

**Published:** 2023-10-02

**Authors:** Sristy Shikha, Mukesh Kumar Jogi, Ruchika Jha, Rana Amit Kumar, Tathagat Sah, Pushpendra Singh, Ritu Sagar, Anuj Kumar, Robin Marwal, Kalaiarasan Ponnusamy, Subhash Mohan Agarwal, R. Suresh Kumar, Nazneen Arif, Mausumi Bharadwaj, Shalini Singh, Pramod Kumar

**Affiliations:** ^1^ Division of Molecular Biology, Indian Council of Medical Research (ICMR)-National Institute of Cancer Prevention and Research (NICPR), Noida, India; ^2^ Amity Institute of Biotechnology, Amity University, Noida, India; ^3^ Department of Biotechnology, Vinoba Bhave University, Hazaribagh, Jharkhand, India; ^4^ Department of Biotechnology, Anugrah Narayan College, Patna, Bihar, India; ^5^ Department of Chemical Engineering and Biotechnology, Beant College of Engineering and Technology, Gurdaspur, Punjab, India; ^6^ Department of Biotechnology, Central University of Haryana, Mahendergarh, Haryana, India; ^7^ Biotechnology Division, National Centre for Disease Control, Delhi, India

**Keywords:** SARS-CoV-2, COVID-19, mutation, omicron, epitopes, antigenic determinant, neutralizing antibodies

## Abstract

The SARS-CoV-2 omicron variants keep accumulating a large number of mutations in the spike (S) protein, which contributes to greater transmissibility and a rapid rise to dominance within populations. The identification of mutations and their affinity to the cellular angiotensin-converting enzyme-2 (ACE-2) receptor and immune evasion in the Delhi NCR region was under-acknowledged. The study identifies some mutations (Y505 reversion, G339H, and R346T/N) in genomes from Delhi, India, and their probable implications for altering the immune response and binding affinity for ACE-2. The spike mutations have influenced the neutralizing activity of antibodies against the omicron variant, which shows partial immune escape. However, researchers are currently exploring various mitigation strategies to tackle the potential decline in efficacy or effectiveness against existing and future variants of SARS-CoV-2. These strategies include modifying vaccines to target specific variants, such as the omicron variant, developing multivalent vaccine formulations, and exploring alternative delivery methods. To address this, it is also necessary to understand the impact of these mutations from a different perspective, especially in terms of alterations in antigenic determinants. In this study, we have done whole genome sequencing (WGS) of SARS-CoV-2 in COVID-19 samples from Delhi, NCR, and analyzed the spike’s mutation with an emphasis on antigenic alterations. The impact of mutation in terms of epitope formation, loss/gain of efficiency, and interaction of epitopes with antibodies has been studied. Some of the mutations or variant genomes seem to be the progenitors of the upcoming variants in India. Our analyses suggested that weakening interactions with antibodies may lead to immune resistance in the circulating genomes.

## Introduction

In November 2021, an omicron variant, BA.1, emerged in South Africa and rapidly spread across the globe, becoming predominant worldwide, including India. Omicron spike protein binds to ACE2 with an affinity similar to the Delta variant despite having many mutations in it (1). However, it shows remarkable antibody evasion as compared to Delta (2). A pseudovirus containing an omicron spike lost binding affinity to NTD (spike)- directed antibodies is probably due to the Δ144-145 deletion in the spike. Similarly, a significant decrease in binding affinities to RBD-directed antibodies (eg. ab1, ab8, and S2M11) is likely due to the other mutations accumulated in respective epitopes in RBD-Omicron (3) (4). Later, Omicron subvariant BA.2, with mutation L452 in the S protein, dominated worldwide, including India, due to its higher rate of transmission and immune evasion in BA.1 infected individuals (5, 6). Two important sub-lineages of omicron emerged from BA.2, one N460K substitution in BA.2.75 and two L452R and F486V substitutions in BA.4 & BA.5 (7). The BA.2.75 sublineage first emerged in India and showed a growth advantage during the surge in the subcontinent. It further accumulated mutations (R346T, F486S, D1999N) and evolved into BA.2.75.2 which dominated in India (7, 8). The neutralization efficiency of BA.2.7.5.2 sub-variants significantly diminished against the antibodies of triple-vaccinated individuals and most of the therapeutic mAbs (9). Along with immune evasion both in vaccinated (one, two, or three doses) and infected individuals, the BA.2 sublineage retains a strong binding affinity with the host ACE2 receptor. The Y505 reversion in the Omicron subvariant was uncommon, and its implication other than ACE2 binding is not known. A G339D mutation was observed in the Delta variant, but G339H was not observed in other prevalent variants, and its implications were not clear (10). The R346T mutation in BA 2.75 and BF7 lineages was implicated in immune evasion, however, the role of R346N in immune evasion and antigenic alterations is not known. BA2.75 or BA2.75.2 spread quickly because it was able to get around the immune system better and stayed strongly attracted to its entry receptor, ACE2.

In this study, we report the results of genome sequencing conducted on the SARS-CoV-2 variants identified in Delhi. Our research reveals that the virus has important mutations, such as Y505 reversion, G339H, and R346T/N, which could change the virus’s immunogenic determinants.

## Materials and methods

### Details and processing of the samples

ICMR-National Institute of Cancer Prevention and Research (ICMR-NICPR) NOIDA has a High Throughput Viral Diagnostic Laboratory (HTL) facility to test SARS-CoV-2 samples from Delhi and some UP regions of India. Nasopharyngeal/oropharyngeal samples (NPS/OPS) were collected from these regions in a viral transport medium (VTM) and transferred to ICMR-NICPR. 24 specimens were included in the study, 14 specimens represented vaccination and 10 specimens were non-vaccinated (TS1). To comprehend the exposure of various SARS-CoV-2 variants, a selection of these samples was put through whole-genome sequencing (WGS). The major inclusion criteria for WGS (RT-PCR) were positive SARS-CoV-2 screening and a real-time PCR cycle threshold value (Ct value) of ≤ 30. To avoid any kind of dissemination of SARS-CoV-2 to healthcare personnel, samples were initially thermally inactivated at 56°C for 30 minutes.

### RNA extraction and RT-qPCR

The viral genomic content of SARS-CoV-2 was isolated from NPS/OPS specimens using 200 μL of the VTM sample from GB Pure Coronavirus RNA Isolation kit (Genuine Biosystem). This extraction protocol is based on magnetic beads for easy and fast isolation of RNA in less than 30 minutes. Nucleic RNA was isolated as directed by the manufacturer and eluted in 40 μl of elution buffer.

The inactivated samples underwent RT-qPCR analysis using a COVID-19 RT-qPCR kit (GENES2ME VIRALDTECT-II) in order to determine the viral load, as previously reported (11). SARS-CoV-2 containing samples/positive samples having Ct values ≤ 30 for RdRp Gene, E Gene, and N Gene (**Supplementary Figure S1**) were subsequently used for whole genome sequencing (WGS) of SARS-CoV-2 (11).

### Whole genome sequencing

SuperScript™ VILO™ Master Mix (TFS) was used for cDNA synthesis from each isolate as follows: 5X VILO^™^ Reaction Mix 2 μl; 10X SuperScript^™^ Enzyme Mix 1 μl; and DNase-treated total RNA (10-12 ng) ≤ 7 µl mixed properly in a MicroAmp™ Optical 96 well reaction plate (0.2ml), sealed and centrifuged. The sealed plate was loaded into the thermal cycler for cDNA synthesis, setting the program as follows: 42°C for 45 minutes, 85°C for 5 min and hold on 10°C. The rest of the protocol was followed as per the manufacturer’s instructions (details mentioned in supplementary information). The sequencing reads were aligned with the NCBI SARS-CoV-2 Reference Genome using Torrent Suite v. 5.18.1. Several plugins were used to learn more about the genetic differences found in the data. These included Coverage Analysis (v1.3.0.2), Variant Caller (v5.16.0.0) with the default settings of “Generic-S5/S5XL (540)-Germ Line-Low Stringency,” and COVID19AnnotateSnpEff (v1.3.0.2), a plugin made for SARS-CoV-2 that can predict the effects of a base substitution. To confirm the accuracy and consistency of the nucleotide calls, Integrative Genomic Viewer_2.14.1 (IGV) software was employed to visualize the TVC (torrent variant caller) Bam files for each sample. The genome sequences of all the SARS-CoV-2 samples were shared in GISAID and NCBI (**Supplementary Table TS2**).

### Sequence analyses and genetic relatedness (phylogeny)

**Figure 1:**
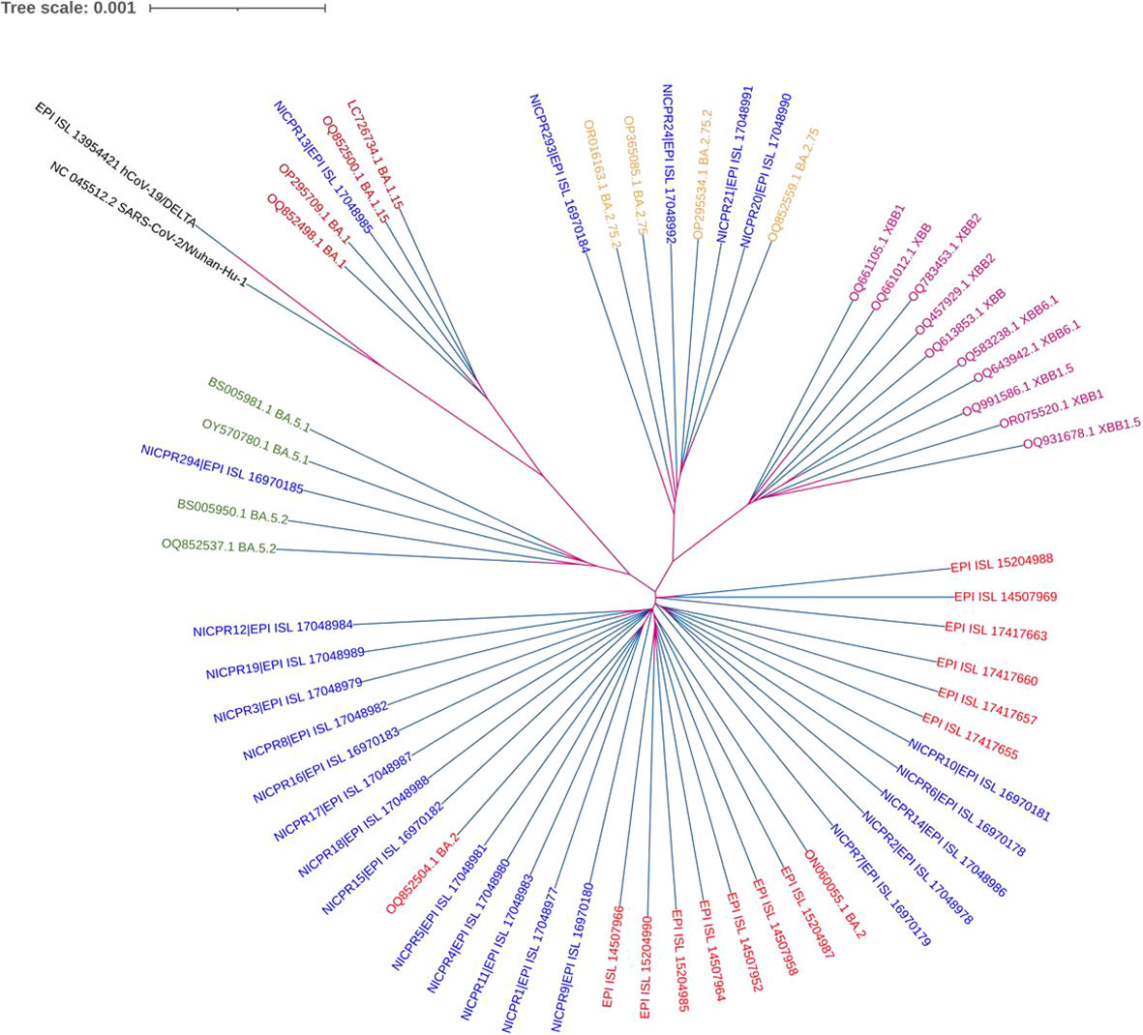
Phylogenetic analysis of WGS of SARS-CoV-2 along with other variants. NICPR samples are indicated in different colors with highlighted background for visualization with their Omicron subvariants in the same color. The multiple sequence aligned with the MEGA 11 and nexus file was used to construct and visualize the phylogenetic tree (maximum likelihood method) using the iTOL software. Default settings have been used in MEGA 11 and iTOL analysis.

The nucleotide sequences of all samples were analyzed using the BAM (Binary Alignment Map) file in IGV software (IGV_2.16.1) for whole genome analysis. All 24 sequences were aligned to the SARS-CoV-2 reference genome (NC 045512.2) using MEGA 11 software (12). An aligned file was downloaded in the nexus format to construct a phylogeny tree by using iTOL (**Figure 1**) (13). Additionally, lineage assignment was carried out using Pangolin (14). The multiple sequence alignment file was visualized for the phylogenetic analysis using the iTOL software. The same nexus file was used to construct the phylogenetic tree using the maximum likelihood method and visualization was carried out by the iTOL. Default settings have been used in MEGA 11 and iTOL analysis. The tree was made with appropriate parameters for optimal visualization.

### Mutation analyses in spike protein

Corrected nucleotide sequences were translated to amino acid sequences using the Expasy Translation tool (https://web.expasy.org/translate/).

Mutation analysis was performed for all structural proteins of SARS-CoV-2. For this, these sequences were aligned using Multalign version 5.4.1 software. The default setting of Gap weight of 12, Gap length-weight 2, Consensus level high (90%) and low (50%) was selected. The result was generated in image format (http://multalin.toulouse.inra.fr/multalin/) (15).

### Pathogenicity analysis

PredictSNP tool was used to predict the pathogenicity of all mutations (https://loschmidt.chemi.muni.cz/predictsnp1/) (16). This server contains the prediction algorithms of several programs, such as MAPP, PolyPhen1 and PolyPhen2, SIFT, SNAP, and PANTHER these were utilized to achieve a consensus pathogenicity score. Thus, it provides a high degree of accuracy due to the consensus technique.

### Prediction of glycosylation alterations in spike

NetNGlyc-1.0 Server Output was used for the prediction of N glycosylation site (https://services.healthtech.dtu.dk/services/NetNGlyc-1.0/) (17) with a threshold potential of 0.5. The server uses Artificial Neural Network (ANN) and predicts the site based on the NXS/T sequence. The server also calculates the glycosylation ‘potential’ which is the average of nine ANN.

### Epitope mapping to identify conserved and altered immunogenic regions in spike

The selection of alleles was done from the available literature that was based on the association of alleles with COVID-19 confirmed cases across the world (18–20). For MHC class I, the following sixteen HLA alleles A02:01, A11:01, A24:02, B07:02, B08:01, B13:02, B18:01, B35:01, B40:06, B46:01, B51:01, B52:01, C01:02, C04:01, C6:02 and 07:01 were used. Cytotoxic T cell (CTL) epitope prediction, NetMHCPan 4.1 was used, which is based on an artificial neural network (ANN). Sixteen selected HLA class I molecules were selected for analysis.

### B cell epitope prediction

B-cell epitope was predicted using ABCpredtool https://webs.iiitd.edu.in/raghava/abcpred/index.html server (21). This prediction tool is based on an artificial neural network. Default parameters of this prediction tool; window length/epitope length (16 amino acids) and threshold of 0.51 were used for prediction.

### Evaluation of impact of mutations through structural analysis

The complex structure of spike protein of SARS CoV-2 complex with human ACE 2 (hACE2) receptor was downloaded from the protein data bank (PDB ID: 6lzg) and used for mapping the mutations (22). Similarly, the complex structure of spike protein with antibodies S309 (PDB ID: 7xsw); Complex structure of gamma P.1 variant spike protein with Fab 4A8 (PDB ID: 8dls), and complex structure of omicron variant spike protein with Fab XGv282 (PDB ID: 7we7) were downloaded from the PDB database. Using the PDB-editor stand-alone tool, complex structures were changed to focus on the surface area of the spike protein with hACE2/antibody. The mutated residues were mapped on the complex structures using PyMOL.2(https://pymol.org/2/). For changing a specific residue and identifying the bonds in the structure “mutagenesis tool” of PyMOL was used.

### Docking of spike-RBD with ACE-2 receptor

Protein-protein docking was used to determine the effect of mutation on the binding affinity of RBD to ACE-2 receptor and antibody against RBD. The sequences of the RBD region were obtained from the sequencing data of individual samples having mutations in the RBD region. The structure was predicted by homology-based modeling using the I-TASSER (Iterative Threading ASSEmbly Refinement) server (https://zhanggroup.org/I-TASSER/about.html). Protein-protein docking was performed by docking the predicted structure of RBD with ACE-2 receptor structures (PDB ID - 1R42) using the HDOCK server (http://hdock.phys.hust.edu.cn/) and the structure of the complex, docking scores, and confidence scores were obtained.

The interaction of RBD regions (with mutation; already predicted by I-TASSER) with antibody 4A8 (PDB ID - 8DLS) was predicted by docking using the HDOCK server; and the structure of the complex, docking scores and confidence scores were obtained.

## Results

### Genetic relatedness and variant analyses

The genome sequences that have been compiled have been formally submitted to the Global Initiative on Sharing All Influenza Data (GISAID). The corresponding accession numbers, together with their respective sub-lineages, have been mentioned in the **Supplementary Table (TS-2)**. A phylogenetic tree based on the WGS of all 24 samples of SARS-CoV-2 is shown in **Figure 1**. The genomic coverage for all samples included in the phylogenetic tree analysis was ≥98%. In addition to the 24 samples, we evaluated the sequences of spike protein of several globally circulating variants to gain a more in-depth knowledge of viral infections (**Supplementary Figure S2**). The lengths and branches in the cladogram represent the evolutionary relatedness of the consensus sequence and samples. These sequences were grouped in 4 clades representing Omicron sublineages as BA.1.15, BA.2, BA.2.75.2 and BA.5. Maximum genomes (75%) belong to BA.2, followed by 8.3% of BA.2.75, 8.3% BA.2.75.2, 4.2% BA.1.15 and 4.2% BA.5. The genomes grouped in BA2.75.2 were more genetically related to XBB lineage.

### Analyses of mutations in structural proteins

All structural proteins, namely spike, nucleocapsid, membrane, and envelope proteins, had a total of 48, 9, 6, and 2 mutations, respectively. The frequency of each mutation within the samples is depicted in **Figure S3** (**Supplementary Material**).

Two mutations were detected in the envelope protein, namely at locations T9I (all samples) and T11A (only four samples). The aforementioned mutations have been documented to exhibit a correlation with the ion-selectivity of envelope channels and a modification in pH sensitivity. Additionally, they have been associated with a decrease in cytokine production and cell death. The altered repercussions resulting from this mutation may perhaps account for the diminished efficiency in the release of the Omicron variant and the subsequent decrease in cellular damage (23).

The glycosylation pattern of membrane proteins can be influenced by mutations occurring at D3N (sample NICPR 294) (24) also found that the presence of D3G (sample NICPR 13) at the 3-8 position can be linked to the N-myristoylation site. The *In-silico* investigation of mutations Q19E and A63T exhibits uncertainty, as some analyses indicate that these mutations lead to the destabilization of the membrane protein’s structure, while other analyses imply that they contribute to its stabilization.

The nucleocapsid protein encompasses nine mutations, there is a mutation P13L and deletion of ERS at positions 31, 32, and 33 which are present at the N-terminal domain of the protein. This domain helps in modulating the RNA binding, and phase separation; thus, mutation at these sites affects the RNA binding. Mutations at positions 203 & 204, were observed in all the samples present in the linker region. At position 413, due to the change in codon, from AGT to CGT, alteration in amino acid, from Serine to Arginine, was seen in all the samples except one (i.e. NICPR13). This mutation is present in the C-terminal and it has no evidential effect on dimerization (25). F307L and D343G are unique mutations in samples NICPR293 and NICPR13 respectively, for which experimentation is required to establish their functional attributes.

**Figure 2:**
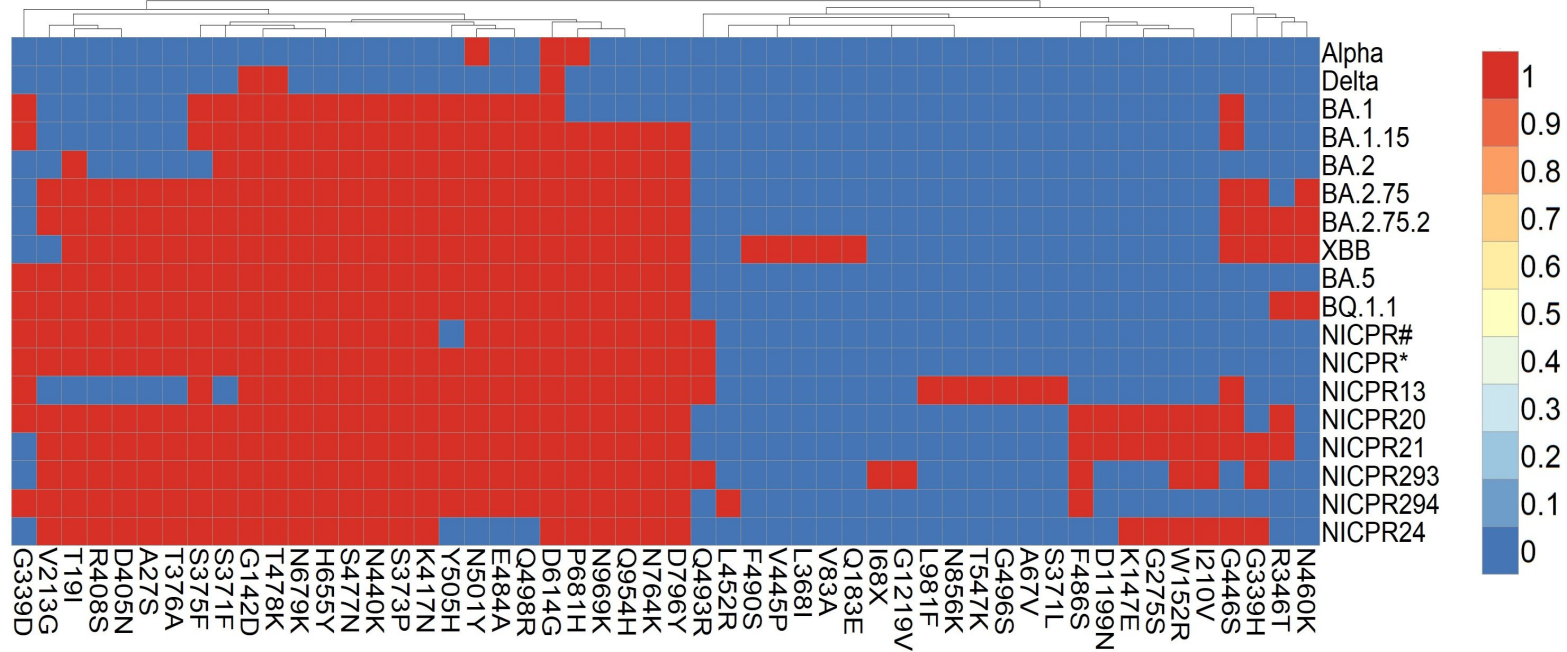
Heat map of mutations in spike glycoprotein prevalent in major variants of SARS-CoV-2. Red boxes represent mutation in the SARS-CoV-2 variants and genomes included in the study, blue boxes represent absence of amino acid mutation in the spike glycoprotein. NICPR# represents shared set of mutations in genomes of NICPR1, NICPR4, NICPR5, NICPR6 and NICPR 11. NICPR* represents shared set of mutations in genomes of NICPR2, NICPR3, NICPR7, NICPR8, NICPR9, NICPR10, NICPR12, NICPR14, NICPR15, NICPR16, NICPR17 and NICPR18.

The Omicron variant has a maximum number of mutations among the other Variants of concern. Spike being the largest structural protein, is highly mutated among all structural proteins. Similar to earlier observations, we also obtained mutations for BA.1, BA.2, and BA.5 (**Figure 2**).

The genomes identified as NICPR1, NICPR4, NICPR5, NICPR6, and NICPR11 exhibit a shared profile of mutations or single nucleotide polymorphisms (SNPs) in spike. Their genetic lineage corresponds to the omicron variant BA.2, with the presence of additional SNPs (T19I, A27S, V213G, S375F, T376A, D405N, R498S, and Q493R) that distinguish them from the canonical lineage. Similarly, another set of samples denoted as NICPR2, NICPR3, NICPR7, NICPR8, NICPR9, NICPR10, NICPR12, NICPR14, NICPR15, NICPR16, NICPR17, and NICPR18 shared the identical set of SNPs (**Figure 2**). Their genetic lineage aligns with the omicron variant BA.2, characterized by the inclusion of additional SNPs. However, noteworthy differences arise in the additional SNPs identified in these specimens, aligning them more closely with the subsequent XBB genetic lineage. Most of these mutations are related to the immune escape by reducing the neutralization activity of antibodies (26). In the later part of the study, we focused on the spike protein only.

### Impact of mutations predicted from PredictSNP

**Figure 3:**
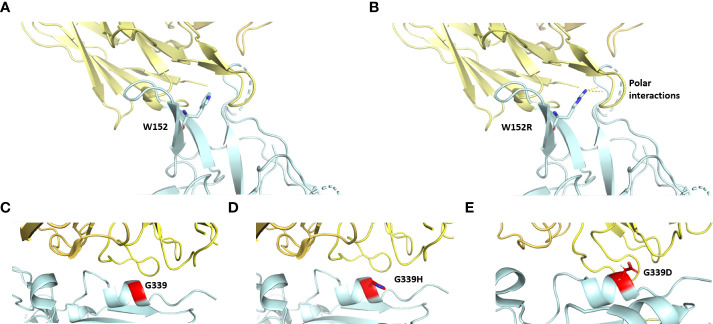
The interfacial region of spike protein in cyan and antibody in yellow is shown. Interaction of spike protein’s residue152 before **(A)** and after mutation **(B)** to the antibody (PDB ID: 8dls. Interaction of spike protein’s residue G339 with antibody before **(C)** and after mutation G339H in Delta variant **(D)** and G339D in omicron variant **(E)** (PDB ID: 7xsw).

The impact and nature of single nucleotide polymorphisms (SNP) were examined using PredictSNP for 48 amino acids (**Supplementary Table, TS-3**), which revealed that most of the mutations (i.e.,41) are neutral in nature whereas seven are predicted to be deleterious (**Tables 1**, **TS-3**). Out of the total of 48 mutations, a subset of 13 mutations were not subjected to annotation and are presented in **Table 1**. Out of the seven detrimental mutations discussed in TS-3, it is worth noting that five have been identified and documented for their involvement in immune evasion and increased susceptibility to infection. Two unique mutations (W152R and G339H) were observed that were not annotated according to PredictSNP. The impact of W152R mutation (NTD region) was studied *in silico* and found to interact with class III antibodies (**Figure 3A**). There is the formation of extra-polar interactions due to the presence of arginine residue (**Figure 3B**). We looked at the G339H and G339D mutation of the RBD region that falls in the group E epitopes how it affects the way ACE2 binds and how the immune system does not recognize it (**Figures 3C–E**).

**Table 1:** Mutation analyses in spike protein and their expected implications.

S.No.	Wild residue	Position	Target residue	PredictSNP prediction	PredictSNP expected accuracy
1	T	19	I	NEUTRAL	0.65307311
2	A	27	S	NEUTRAL	0.82622462
3	K	147	E	NEUTRAL	0.73834499
4	W	152	R	DELETERIOUS	0.60548272
5	I	210	V	NEUTRAL	0.82622462
6	G	257	S	NEUTRAL	0.82622462
7	G	339	H	DELETERIOUS	0.63587604
8	R	346	T	NEUTRAL	0.65307311
9	S	371	F	NEUTRAL	0.73834499
10	N	460	K	NEUTRAL	0.82622462
11	F	486	S	NEUTRAL	0.82622462
12	A	846	S	NEUTRAL	0.65307311
13	D	1199	N	NEUTRAL	0.65307311

### Mutations in the receptor binding motif of spike

**Figure 4:**
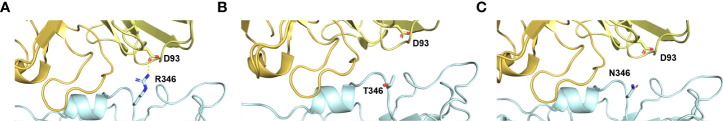
Implications of R346T/N mutations in possible immune evasion. **(A)** the binding of Omicron spike with antibodies specific for class III. **(B, C)** Loss of this interaction in case of mutation R346T/N with D93 of ACE2 receptor.

Mutations at the RBM of the RBD-spike have been identified as Q493R, G496S, Q498R, and Y505H. **Figure 4** also shows that the positions of Q493, G496, and Y505 have reverted. G496 reversion is also observed in the Omicron BA1 lineage. Q493 reversion mutation was observed among genotypes belonging to BA2.75 (NICPR20) and BA2.751(NICPR21). Q493R mutation in Omicron was observed as a compensatory mutation (gain of salt bridge between Omicron-spike and ACE-2) to K417N (loss of salt bridge between Omicron-spike and ACE-2) when compared with Delta-spike (3). The K417N is related to immune escape by decreasing antibody binding. The Q493 reversion would result in the loss of the salt bridge between Omicron-spike and ACE-2. Y505 reversion was only observed in BA2.75 (NICPR20), the Y505 position was also involved in ACE-2 binding through a Hydrogen bond.

### Interaction of RBD domain with ACE-2 receptor and antibodies

The docking score of the RBD domain representative sample of different Omicron variant groups to the ACE-2 receptor has been mentioned in **Table 2**. A lower docking score suggests a higher affinity for binding. The docking scores are computed using our knowledge-based iterative scoring functions, namely ITScorePP or ITScorePR. A lower docking score indicates a higher likelihood of a binding model. The confidence score determines the likelihood that the two molecules will bind: more than 0.7 indicates that it is highly likely; between 0.5 and 0.7 indicates that it is feasible; and less than 0.5 indicates that it is unlikely. In all the samples except sample 16, there is a decrease in docking score as compared to WT-SARS-CoV-2 and Delta, suggesting increasing affinity for binding to ACE-2 receptors. NICPR 19 and NICPR 21 have the highest binding affinity to the receptor.

**Table 2:** The structures of the following samples were predicted from I-TASSER and docking with ACE-2 receptor (PDB ID- 1R42) by HDOCK server.

RBD binding with ACE2 receptor			RBD binding with Antibody		
Sample	Docking score (kcal/mol)	Confidence score	Sample	Docking Score (kcal/mol)	Confidence Score
Wuhan	-252.7	0.8864	Wuhan	-231.57	0.8364
Delta	-257.13	0.895	Delta	-234.80	0.845
NICPR1	-275.29	0.9245	NICPR1	-224.49	0.816
NICPR13	-269.87	0.9166	NICPR13	-229.16	0.8297
NICPR16	-245.49	0.871	NICPR16	-201.85	0.7383
NICPR19	-294.91	0.9478	NICPR19	-261.33	0.9026
NICPR21	-294.33	0.9472	NICPR21	-234.66	0.8446
NICPR293	-272.14	0.92	NICPR293	-249.25	0.8792
NICPR294	-283.32	0.935	NICPR294	-298.75	0.8551

Similarly, RBD interaction with antibodies of samples NICPR1, 13, 16, and 21 are nearly the same, NICPR19 and 293 have less, and NICPR294 has the lowest docking score, indicating the lowest, moderate, and highest binding to the antibodies. 

### Alteration in N-glycosylation sites in spike protein

SARS-CoV-2 and target cell glycosylation both have a significant influence on SARS-CoV-2 infection at various levels. Analyses of N-glycosylation in the spike glycoproteins revealed the presence of 22 sites for glycosylation (**Supplementary Table 3 TS 3**). Except for four sites of N-glycosylation at position 17 (NLTT), 149 (NKSW), 165 (NCTF), and 343 (NATR), the rest were conserved in all the samples (**Table 3**). N glycosylation site at position 17 (NLTT) was only present in one sample representing BA1.15. However, there was a loss of N165 (NCTF) glycan in the sample. This glycosylation site was also absent in the delta variant and only present in BA.1 and BA1.15. A reversal (absence) of the glycosylation at position 17 was observed in BA.2, BA2.75, and BA5 sub-lineages.

**Table 3:** N-Glycosylation sites predicted in spike glycoprotein from different variants.

Position	Site	Delta	BA1	BA1.15	BA2	BA2.75	BA5	Nicpr1	Nicpr9	Nicpr 13	Nicpr20, 21	Nicpr24	Nicpr293	Nicpr294
17	NLTT	_	+	+	–	–	–	_	–	+	–	–	–	–
149	NKSW	+	+	+	+	NKSR	+	+	+	+	NKSR	NKSR	NKSR	+
165	NCTF	+	+	+	+	+	+	+	+	–	+	+	+	+
343	NATR	+	+	+	+	NATT	+	+	–	+	NATT	NATN	+	+

We have further checked the change in glycosylation extent (**Supplementary Table 4**) for positions 149 (NKSW) and 343 (NATR). There are some mutations at nearby residues of the N-glycosylation site that change the potential of glycosylation, such as at position 149 NKSW to NKSR, the potential of glycosylation occurrence is increased to 0.6318 to 0.6946 (nearly 10%) as compared to the WT-SARS-CoV-2 sequence. At another position, 343 NATR to NATT the potential of glycosylation decreases from 0.5671 to 0.5497. For the same position, there is an increase in the glycosylation potential to nearly 10% for sample NICPR293 which has the same residue as the WT-SARS-CoV-2 sequence NATR with some mutations before this region (8 residues before). The interaction of these residues may be responsible for the increase in glycosylation potential.

### Identification of altered and conserved immunogenic regions (epitopes) in spike glycoprotein

In this part of the study, spike proteins of samples NICPR13, NICPR20, NICPR21, NICPR293, and NICPR294 were considered and compared with the reference sequence of SARS-CoV-2 (Wild Type or WT SARS-CoV-2 is used for representation). A large number of epitopes of different affinity were generated upon using the NetMHCPan 4.1 prediction tool, only strong binders to cytotoxic T cells were selected for further analysis. In a similar way for helper T cells, various epitopes in spike were compiled with higher affinity/Strong HLA binders to induce the immune response (shown in **Supplementary Table 6**). The total number of obtained epitopes for CTL, HTL, and B-cell epitopes from all selected samples are summarized in **Table 4** and epitopes are compiled in **Supplementary Tables 5**–**7** respectively.

**Table 4:** Identification of immunogenic regions (epitopes) in spike glycoprotein of the representative variants.

S.No	Samples	CTL	HTL	B cell epitopes
1	WT-SARS-CoV-2	105	158	133
2	NICPR13	98	113	131
3	NICPR20	104	118	128
4	NICPR21	104	116	128
5	NICPR293	103	118	130
6	NICPR294	101	117	129

### Determination of immunogenic regions for cytotoxic T cells

The majority of anticipated epitopes are conserved in the Reference SARS-CoV-2 spike, and the allele-presenting characteristics of all samples are similar. Due to changes in amino acid sequences, some epitopes have changed in amino acid composition in comparison to WT, few epitopes remained only in the WT SARS-CoV-2, and this has also led to the formation of new epitopes. All the epitopes are mentioned in the **Supplementary Table 5**. However, the new epitopes or altered epitopes within the selected samples are mentioned in **Table 5**. These newly formed epitopes may play a role in generating immune escape in SARS-CoV-2-infected individuals.

**Table 5:** Altered immunogenic determinants (epitopes) in the spike for cytotoxic T cells.

SN	Position	Sequence	Samples
1	321	NLCPFDEVFNA	NICPR13 & NICPR294 (328)
2	972	SSVLNDILSRL	WT SARS-CoV-2(976), NICPR20(970), NICPR21(970), NICPR293(968) & NICPR294(968)
3	968	FSRLDKVEAEV	NICPR13
4	1187	AKNLNESLINL	NICPR20 & NICPR21
5	741	LQYGSTQLK	NICPR293(749), NICPR294(749), NICPR13 (742), NICPR20 (751) & NICPR21 (751)
6	21	RTYTNSFTR	NICPR20, NICPR21, NICPR293 & NICPR294
7	166	TYVSQPFLM	WT SARS-CoV-2, NICPR20(164), NICPR21(164), NICPR293(162) & NICPR294(162)
8	157	VYSSANNTF	NICPR13(439), NICPR20(156), NICPR21(156), NICPR293(154), NICPR294(154) & WT SARS-CoV-2
9	200	KPINLGRDL	Only in NICPR294
10	450	YLYRRKSNL	WT SARS-CoV-2 & NICPR13(439)
		YLYRRKSKL	NICPR293(446), NICPR21(448) & NICPR20(448)
11	244/242	YPGDSSSSW	NICPR20 (244), NICPR21(244), NICPR293(242)
12	21	TQLPPTNSF	WT SARS-CoV-2, NICPR13
13	212	VLPQGFSAL	WT SARS-CoV-2
		GLPQGFSAL	NICPR20(210), NICPR21(210), NICPR293(208) & NICPR294(208)

### Determination of immunogenic regions for helper T cells

**Figure 5:**
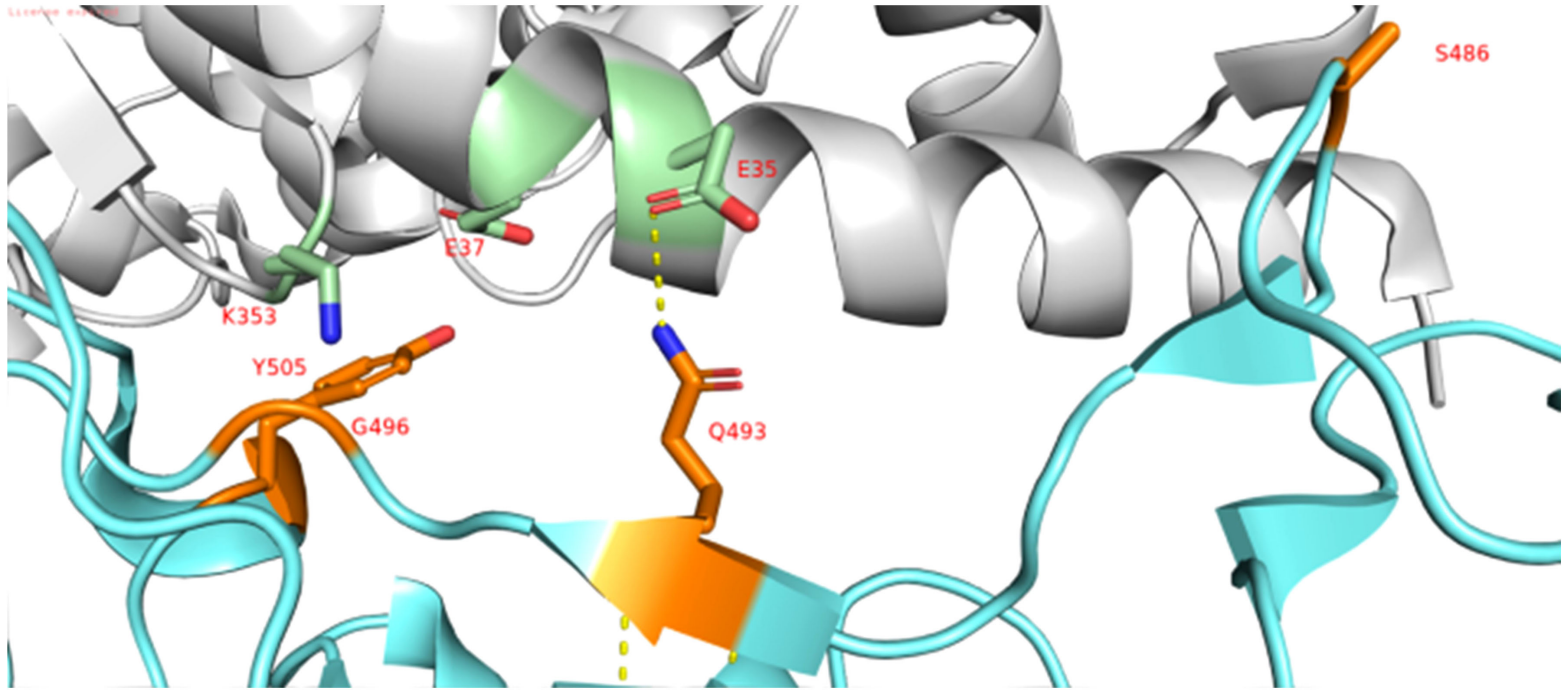
Interaction of RBD domain of Spike in cyan to ACE-2 Receptor in grey (PDB ID: 6lzg). The interacting residues of RBD domain are shown in orange and interacting residues of ACE-2 receptor are shown in green (N atom in selected residues is shown blue and O atom is shown in red).

HTL analysis of spike proteins in these samples’ results suggests that the majority of the epitopes are shared throughout the NICPR samples and exhibit conservation to the wild-type SARS-CoV-2 (**Supplementary Table 6**). New epitopes or altered epitopes within the selected samples are mentioned in **Table 6**. However, few epitopes are unique to BA.1.15 (sample 13), epitopes YSKHTPIIV and FSRLDKVEA. Two epitopes (FVIRGNEVS & IRGNEVSQI) were observed unique to samples 20, 21, 293, and 294. YHKNNKSRM epitope was unique to sample 294. The W152R mutation or the corresponding epitope seems a novel gain of helper T cell epitope (YHKNNKS**R**M) might compensate for the loss of helper T cell epitope due to the R346T/N (FNAT**R**FASV) site (**Figures 5A–C**). The mutation R346T mutation loosens the binding of spike glycoprotein with class three neutralizing antibodies (**Figure 5B**) that is also similar to R346T (**Figure 5C**) The mutation also results in the loss of a potential epitope (FNAT**R**FASV) as evident from the epitope predictions.

**Table 6:** Immunogenic determinants (epitopes) in spike protein for helper T cells (HTL).

SN	Sequence	Samples and Positions
1	FNGLTGTGV	WT SARS-CoV-2 (543), NICPR293 & NICPR294 (538), NICPR20 & NICPR21 (540)
	FNGLKGTGV	NICPR13 (531)
2	FHAISGTNG	NICPR293 (62)
	FHVISGTNG	NICPR13 (65)
3	YSKHTPIIV	NICPR13 (190)
4	FVIRGNEVS	NICPR20(397), NICPR21(397), NICPR293(395), NICPR294(395)
5	FSRLDKVEA	NICPR13 (969)
6	LIVNNATNV	NICPR24 (115)
7	YHKNNKSRM	NICPR293 (140)
8	IRGNEVSQI	NICPR293(397), NICPR294 (397), NICPR20(399), NICPR21(399)
9	FLDVYYHEN	NICPR20(137), NICPR21(137)
	FLDVYYHKN	NICPR293(135), NICPR294(135)

The mentioned epitopes are either unique, or altered, or lost in some variants. The position of each epitope is mentioned in the bracket.

### Determination of immunogenic regions for B cells

In addition to cellular immunity, humoral immunity mediates pathogen elimination in an antibody-dependent manner. To know the putative epitopes, the ABCPred server was used to further scan the SARS-CoV-2 spike protein for linear B-cell epitopes selecting the default parameter. Based on the above criteria, we obtained a total of 132 epitopes. Most of the B-cell epitopes are conserved (nearly 63%), while some are only present in WT-SARS-CoV-2 (Nearly 16%) (**Supplementary Table 7**). Mutations in some epitopes have changed the antigenic score in the determinant (shown in **Table 7**). This could alter the epitope’s interaction with the antigenic determinant, which could aid in SARS-CoV-2 variants’ immunological escape from existing therapies. Unlike CTL and HTL-predicted epitopes, no new epitopes were discovered in our sample. Some overlapping epitopes are also present in WT-SARS-CoV-2 as well as our sequences, which can associate with some alterations in antigenic/immunogenic potential and may contribute to immune evasion (**Table 7**). An epitope profiling study performed (27) on isolated epitopes from SARS-COV-2 infected individuals, has found overlapping regions with our predicted epitope findings, such as positions 19, 25, 257, and 547. Another study performed by found a conserved epitope 407VRQIAP412 (Sample NICPR-13) epitope and highly variable peptide/region among the SARS-CoVs 473YQAGSTP479 (Samples NICPR-13, 21, 293 & 20) (28).

**Table 7:** Altered immunogenic determinants (epitopes) in spike for B cells.

S.No.	Position	Epitope Sequence	Samples	Score
1	257	GWTAGAAAYYVGYLQP	WT SARS-CoV-2, NICPR294(252), NICPR13(245)	0.95
		SWTAGAAAYYVGYLQP (254)	NICPR21(254), NICPR293 (252), NICPR20(254)	0.94
2	245	HRSYLTPGDSSSGWTA	WT SARS-CoV-2, NICPR294(240), NICPR13(233)	0.92
		HRSYLTPGDSSSSWTA	NICPR21(242), NICPR293(240), NICPR20(242)	0.89
3	1206	YEQYIKWPWYIWLGFI	WT SARS-CoV-2, NICPR13(1194), NICPR20(1203), NICPR21(1203) & NICPR294(1201)	0.89
		YEQYIKWPWYIWLVFI (1201)	NICPR293	0.91
4	464	FERDISTEIYQAGSTP	WT SARS-CoV-2	0.86
		FERDISTEIYQAGNKP	NICPR294(459), NICPR13(452), NICPR21(461), NICPR293(459), NICPR20(461)	0.88
5	406	EVRQIAPGQTGKIADY	WT SARS-CoV-2	0.85
		EVRQIAPGQTGNIADY (394)	NICPR13	0.84
6	391	CFTNVYADSFVIRGDE	WT SARS-CoV-2, NICPR13(379)	0.85
		CFTNVYADSFVIRGNE	NICPR294(386), NICPR21(388), NICPR293(386), NICPR20(388)	0.88
7	200	YFKIYSKHTPINLVRD	WT SARS-CoV-2	0.84
		YFKIYSKHTPINLGRD	NICPR294 (195)	0.79
		YFKIYSKHTPVNLGRD	NICPR20 (197), NICPR21 (197), NICPR293 (195)	0.79
		YFKIYSKHTPIIVREP	NICPR13 (186)	0.89
		FKNIDG**YFKIYSKHTP**	WT SARS-CoV-2(194), NICPR13(180), NICPR20(191), NICPR21(191), NICPR293(189) & NICPR294(189)	0.8
8	847	RDLICAQKFNGLTVLP	WT SARS-CoV-2, NICPR20(844), NICPR21(844), NICPR293(842) & NICPR294(842)	0.83
		RDLICAQKFKGLTVLP	NICPR13 (835)	0.79
9	70	VSGTNGTKRFDNPVLP	WT SARS-CoV-2, NICPR20(67) & NICPR21(67)	0.82
		ISGTNGTKRFDNPVLP	NICPR13 (68) & NICPR293 (65)	0.84
		HXX**SGTNGTKRFDNPV**	NICPR294 (63)	0.85
10	329	FPNITNLCPFGEVFNA	WT SARS-CoV-2	0.82
		FPNITNLCPFDEVFNA	NICPR294 (324), NICPR13 (317)	0.84
		FPNITNLCPFHEVFNA	NICPR21 (326), NICPR20 (326)	0.74
11	360	NCVADYSVLYNSASFS	WT SARS-CoV-2	0.79
		NCVADYSVLYNFAPFF	NICPR294 (355), NICPR21 (357), NICPR293 (355), NICPR20 (357)	0.78
		RKRIS**NCVADYSVLYN**	NICPR13 (343)	0.71
12	19	TTRTQLPPAYTNSFTR	WT SARS-CoV-2, NICPR13	0.79
		VSSQCVNL**TTRTQLPP**	WT SARS-CoV-2 (11),NICPR13 (11)	0.65
13	501	NGVGYQPYRVVVLSFE	WT SARS-CoV-2	0.77
		YGVGYQPYRVVVLSFE	NICPR20 (498)	0.81
14	941	TASALGKLQDVVNQNA	WT SARS-CoV-2	0.76
		TASALGKLQDVVNHNA	NICPR294(936), NICPR13(929), NICPR21(938), NICPR293(936), NICPR20(938).	0.73
15	547	TGTGVLTESNKKFLPF	WT SARS-CoV-2, NICPR20(544), NICPR21(544), NICPR293(542) & NICPR294(542)	0.73
		NGLK**GTGVLTESNKKF**	NICPR13 (532)	0.71
16	484	EGFNCYFPLQSYGFQP	WT SARS-CoV-2	0.73
		AGFNCYFPLQSYGFRP	NICPR293 (479)	0.64
17	953	NQNAQALNTLVKQLSS	WT SARS-CoV-2	0.71
		NHNAQALNTLVKQLSS	NICPR294(948), NICPR13(941), NICPR21(950), NICPR293(948) & NICPR20(950)	0.7
18	445	VGGNYNYLYRLFRKSN	WT SARS-CoV-2	0.71
		KVS**GNYNYLYRLFRKS**	NICPR13(432), NICPR21(441), NICPR293(439), NICPR20(441).	0.78
19	430	TGCVIAWNSNNLDSKV	WT SARS-CoV-2	0.71
		TGCVIAWNSNKLDSKV	NICPR13 (418), NICPR21 (427), NICPR20 (427), NICPR293 (425), NICPR294(425)	0.77
20	25	PPAYTNSFTRGVYYPD	WT SARS-CoV-2, NICPR13	0.7
		TRTQS**YTNSFTRGVYY**	NICPR294 (20), NICPR21 (20), NICPR20 (20), NICPR293 (20)	0.89

The mentioned epitopes are either unique or altered in some variants.

## Discussion

In this study, we performed the WGS of the samples collected at ICMR-NICPR, Noida during the spread of the omicron variant of SARS-CoV-2 during Jan-Feb 2022. The viral genome sequences obtained from the COVID-19 samples have been submitted to GISAID. Most of the samples belong to the BA.2 subvariant. Patients infected with BA.1.1.5 omicron variants were observed to have lower viral load and are associated with less severity of the disease in Southern India. However, patients infected with the BA.1.1 and BA.2 omicron variants were associated with high viral load and severity of the diseases (29).

The virus acquired crucial mutations in spikes in the background of vaccines being used. In the background of vaccines, Covishield (recombinant mRNA encoding spike) and Covaxin (whole virus inactivated) vaccines, India witnessed the emergence of the Delta variant in 2021 in Maharashtra and Delhi (30, 31). The Delta variant was selected for its strong binding of spike to the ACE2 receptor and neutralization resistance against the immune response, including breakthrough infections (3, 31). In the first half of 2022, India again witnessed a peak of COVID-19 associated with the omicron variant, which emerged in South Africa. Omicron spike protein contained a large number of RBD and NTD mutations to retain ACE2 binding affinity similar to the Delta variant with a remarkable antibody evasion (3, 4). The Omicron spike keeps accumulating various novel or reversion mutations to escape immune response both in vaccinated and infected individuals. The spike sequences of the variants included in the study were compared with omicron and other variants of SARS-CoV-2. The genomes NICPR13, NICPR20, NICPR21, NICPR293 and NICPR24 shared the identical SNPs (R346T, G339H and G446S) which were present in the BA.2.75 and BA.2.75.2 variants; however these samples also exhibited some additional mutations (K147E, W152R, I210V, G275S, F486S and D1199N). Some of these mutations were present in the XBB variant which emerged in late 2022. A pivotal observation is that during January 2022, these specimens circulated as intermediate or progenitor to the emerging XBB variations. A notable SNP alteration, G339D, was identified in these specimens. This specific SNP underwent a subsequent alteration to G339H, observed not only in the XBB lineage but also in the variants BA.2.75 and BA.2.75.2.

Some of the mutations in our analysis seem deleterious, while others are neutral with respect to their implications for the host. The mutations W152R and G339H might help in immune escape and binding to the ACE-2 receptor, respectively, thus facilitating viral transmission. A study by Kubik et al. has also observed that this tryptophan (W152) mutation has different amino acids at different timescales and geographical regions (32). This (W152) plays a critical role in interaction with antibodies and mutation alters the interaction and promotes immune escape. Likewise, G339 is a major interacting amino acid to the ACE-2 receptor, and G339D mutation is known to be involved in immune escape (10). The binding affinity of the antibodies (eg. VIR-7831) against group E epitopes with omicron spike is significantly hampered by the G339D mutation (10). Mutation of G339D to G339H inflicts fusogenicity and thus enhances the infection rate to 44-fold (33). It also permits BA.2.75 to escape the host’s effective neutralizing antibody response generated against different RBD epitopes. Glycosylation plays an important role in viral entry and infection. Most glycosylation deletions are less deleterious, however, a combination of N331 and N343 (present in RBD domain) deletions significantly lowers the viral entry and ultimately impacts infectivity (34). This site is also important for immune recognition, which may have given the selection pressure to switch from NATR (343) to NATT/NATN. N165 glycan is crucial in controlling the switch of conformational transition between ‘Up’ and ‘Down’ states of the RBD (35). The N165Q mutant was observed to be more sensitive to RBD-directed MAbs (34, 35). Loss of N165 glycan and gain of N17 glycan in the sample representing BA1.15 may be important in compensating either in resistance to antibody neutralization or controlling conformational switch of the RBD states. The processing of high mannose to complex N-glycan was decreased at N165, and N343 in the omicron variant whereas the N-glycan process at most other sites across the variants is conserved (36). The change in glycan shielding would have implications for spike-mediated viral functions.

In the case of CTL and HTL epitope mapping, mutations in the spike alter the antigenicity parameter of these epitopes and interact with antibodies, thus facilitating immune escape. The new epitopes or changes in the antigenic determinants might be a result of the selection pressure exerted by the host immune response. Our *in silico* data (R346T/N) along with previous studies on neutralization assays suggest this mutation helps in immune evasion in BA 2.75 and BF7 lineages of Omicron (37). This mutation loosens the binding of spike glycoprotein with class three neutralizing antibodies thus enhancing the escape from neutralizing antibodies (34). The mutation also results in a loss of a potential epitope (FNAT**R**FASV) as evident from the epitope predictions.

This reversion would strengthen the binding of the spike with the ACE-2 receptor. Y505 along with the T470-T478 loop are vital binding determinants of viral spike to ACE-2 (38). Y505H mutation was observed in Omicron, resulting in the loss of an H-bonding of the omicron spike with E37 residue of ACE-2 (3). Moreover, Y505 reversion also results in a gain of CTL epitope (VG**Y**QPYRVV) with strong binding affinity to HLA. Some mutations like F486S and R493Q in spike which were also observed in the XBB variant lower the equilibrium constant (KD) value and may reflect in a modest loss of binding affinity with ACE2 (39). Y505 reversion in the spike may compensate for the lower binding affinity with ACE-2 by establishing the H-bond. An immunodominant CTL epitope NYNYLYRLF in spike from COVID-19 patients from Europe and USA was also shared in B-cell epitope (KVSG**NYNYLYRLF**RKS) in some of the genomes (40, 41). Some other immunodominant T cell epitopes reported earlier from Western countries were shared fully (CVADYSVLY) or partially (eg. B44-AEV in spike) in the genomes from our study (41, 42).

Several point mutations (R339H, R346T, N460K, and F486S) which were observed in various SARS-CoV2 variants including the most alarming variants (BF7, BQ1, and XBB) have been reported to be implicated in providing viral resistance to various neutralizing antibodies (37, 39). The gain of novel or reversion mutations in the Omicron spike, allows the virus to escape the host immune response with strong binding affinity to the ACE2 receptor to drive its spread. Therefore, a track of spike mutations and their associated antigenic alterations would be of great importance in designing future vaccine strategies to combat the ongoing pandemic.

## Conclusion

Immune escape mutations were observed in vaccinated and infected individuals from the Delhi region during the Omicron wave. There are lots of mutations that have accumulated in the omicron and its sub-variants. Some mutations observed in spike (Y505 reversion, G339H, and R346T/N) are involved in high binding affinity with the ACE2 receptor, change in the predicted epitopes, and altered binding affinity with MAbs, respectively. The mutations involved in alterations in the epitopes and binding with antibodies may have a role in immune evasion. The mutation involved in strong binding affinity to ACE2 may have implications in viral entry to host cells. There is a need for *in-vitro* and *in-vivo* experiments to support the direct implications of the mutants in higher infectivity and immune evasion.

## Data availability statement

The datasets presented in this study can be found in online repositories. The names of the repository/repositories and accession number(s) can be found in the article/**Supplementary Material**.

## Ethics statement

The studies involving humans were approved by Institutional Ethics Committee, ICMR-NICPR, Noida. The studies were conducted in accordance with the local legislation and institutional requirements. The ethics committee/institutional review board waived the requirement of written informed consent for participation from the participants or the participants’ legal guardians/next of kin because retrospective and anonymized COVID-19 samples were used for the study.

## Author contributions

PK: conceptualization. SSh, MJ, and PK: methodology. AK, SSh, MJ, RJ, RAK, TS, PS, and RS: formal analysis. PK, SSh, MJ, RM, KP, SA, and SS: draft preparation and review. RSK and NA contributed to providing the samples. All authors contributed to the article and approved the submitted version.
