# Supplementary material for: Genome sequencing of SARS-CoV-2 omicron variants in Delhi reveals alterations in immunogenic regions in spike glycoprotein
Source: Front Immunol. 2023 Oct 2;14:1209513. doi: 10.3389/fimmu.2023.1209513 (PMC10577267; doi:10.3389/fimmu.2023.1209513)
Supplement: Supplementary file 1 [file DataSheet_1.docx]

**Supplementary materials**

**Methods**

**c-DNA synthesis and Library preparation**

A library of selected isolates has been synthesized using the two primer pool concepts as follow (per sample): 4.5 µl of 5X Ion AmpliSeq™ HiFi Mix, 10 µl cDNA and 3.5 µl NFW added in 1.5 ml tube and dispensed 8 µl in 21 wells of two PCR plates and then added the 2 µl of primer pool 1 into the 21 wells of first plate, and 2 µL of primer pool 2 to the 21 wells of second plate for a total of 10 µL in each well. To amplify the target regions (Pool primers), thermal cycling was performed using the following conditions: enzyme activation for 2 min at 98^0^ C, followed by 16 cycles at 98^0^ C for 15 s and 60^0^C for 4 min.

Amplified products have been combined from two plates into a new PCR plate and the final volume at this stage was 20 µL. Partial digestion of amplicons has been performed by the adding of 2 μL of FuPa reagent to each amplified sample and mixture was incubated at 50^0^ C for 10 min, 55^0^ C for 10 min and 60^0^ C for 20 minutes. To ligate the adapters to the amplified products 2 µL of diluted Ion Xpress™ Barcode Adapters together with 4 µL of Switch Solution and 2 µL DNA Ligase were added. The samples were then incubated for 30 min at 22^0^ C, 5 min at 68^0^ C, and 5 min at 72^0^ C. After ligation, purification of each library was performed with the Agencourt™ AMPure™ XP Reagent (Beckman Coulter, Brea, CA, USA) and amplified with 50 µL of Platinum™ PCR SuperMix HiFi and 2 µL of Library Amplification Primer Mix using the following conditions: 2 min at 98 ◦C, 5 cycles of 15 s at 98 ◦C and 1 min at 64 ◦C. All the reactions were performed in an Applied Biosystems™ Veriti™ 96-Well Thermal Cycler (Thermo Fisher Scientific). Purification of amplified barcode libraries was done with Agencourt™ AMPure™ XP Reagent (Beckman Coulter), then this purified amplicons were eluted in 50 μL of TE buffer, and quantified by Qubit™ ds DNA HS Assay Kit in Qubit™ Flex Fluorometer. Barcoded libraries were diluted to ~100 pM, and added to the Ion Chef™ library sample tube, and then loaded onto the Ion Chef™ System (Thermo Fisher Scientific) for emulsion PCR, enrichment, and loading onto the Ion S5 540 chip. After the completion of the run, the chip loaded for the next-generation sequencing in the Ion GeneStudio™ S5 System with the use of Ion 540™ Kit-Chef and Ion 540™ Chip Kit. Sequencing results were analyzed in Torrent Suite™ Software with SARS-CoV-2 plugins: variant Caller, SARS-CoV-2 coverage analysis, and IRMA report with standard configuration.

**Phylogeny based on Spike protein**

The resulting amino acid sequences of surface glycoproteins or spike proteins were utilized to perform evolutionary analyses in MEGA11 considering the Wuhan protein accession ID (GenBank: YP_009724390) as reference using the Neighbor-Joining method. The branch lengths of the tree were drawn to scale and are in the same units as the evolutionary distances used to infer the phylogenetic tree. The evolutionary distances were calculated using the Poisson correction method (16) and are expressed in units of the number of amino acid substitutions per site. The phylogenetic analysis included representative amino acid sequences of spikes from various variants of concern (VOCs) of SARS-COV-2, Wuhan (alpha), Delta and Omicron. All ambiguous positions were removed for each sequence pair using the pairwise deletion option. To assess the statistical robustness of the constructed phylogenetic tree, a bootstrap replication of 1,000 cycles was performed. The reference sequence of spike (Wuhan) contains 1273 amino acids. Due to deletion, insertion of some nucleotides, there is a change in the amino acid sequence in the subsequent variants. Aligning all these sequences with reference Wuhan sequence, the final dataset consisted of a total of 1275 positions.

**Table TS1:** Vaccination and clinical status of the samples involved in the study: N-Not, Y-Yes, NV-Non vaccinated.

| **Sample ID** | **Age (Years)** | **Gender** | **Previous Infection** | **Symptoms** | **Vaccine Status** | **Hospitalization** |
| --- | --- | --- | --- | --- | --- | --- |
| NICPR01 | 50 | Male | Y | Fever | 1 dose | N |
| NICPR02 | 25 | Male | N | Fever | NV | N |
| NICPR03 | 21 | Female | N | Fever | NV | N |
| NICPR04 | 30 | Male | Y | Fever | 1 dose | N |
| NICPR05 | 53 | Male | Y | Cold, Fever, Cough | 1 dose | N |
| NICPR06 | 27 | Female | N | Cough, fever | NV | N |
| NICPR07 | 75 | Female | N | Fever, Body ache | NV | N |
| NICPR08 | 26 | Female | N | Fever | NV | N |
| NICPR09 | 14 | Male | N | Cough, cold | NV | N |
| NICPR10 | 15 | Male | Y | Cold, Fever, Cough | 1 dose | N |
| NICPR11 | 50 | Male | Y | Fever | 1 dose | N |
| NICPR12 | 40 | Female | Y | Sour throat | 1 dose | N |
| NICPR13 | 35 | Male | Y | Fever, Body pain, Throat infection | 1 dose | N |
| NICPR14 | 30 | Female | Y | Fever | 1 dose | N |
| NICPR15 | 11 | Female | N | Cough, Cold | NV | N |
| NICPR16 | 67 | Male | N | Fever, Cough, Cold, Breathing Issue | 1 dose | N |
| NICPR17 | 18 | Male | N | Fever | NV | N |
| NICPR18 | 28 | Female | N | Fever | NV | N |
| NICPR19 | 28 | Male | Y | Cold, Fever, Cough | 1 does | N |
| NICPR20 | 26 | Male | Y | Fever | 1 dose | N |
| NICPR21 | 20 | Female | N | Fever, Cough, Cold, Breathing Issue | NV | N |
| NICPR293 | 30 | Female | N | Cold, Fever, Cough, Body pain | 3 dose | N |
| NICPR294 | 32 | Male | Y | Cold, Fever, Cough | 3 dose | N |
| NICPR24 | 40 | Male | Y | Cold, Fever, Cough | 3 dose | N |

**Results:**

In addition to the 23 samples, we evaluated the sequences of several globally circulating variants to gain a more in-depth knowledge of viral infections. The lengths and branches in the cladogram represent the evolutionary relatedness of the consensus sequence and samples. The WGS were grouped in 4 clades representing Omicron sublineages as BA1.15, BA2, BA2.75.2 and BA5. All the sequences have been submitted in GISAID and their accession numbers are mentioned in table ST1.

**
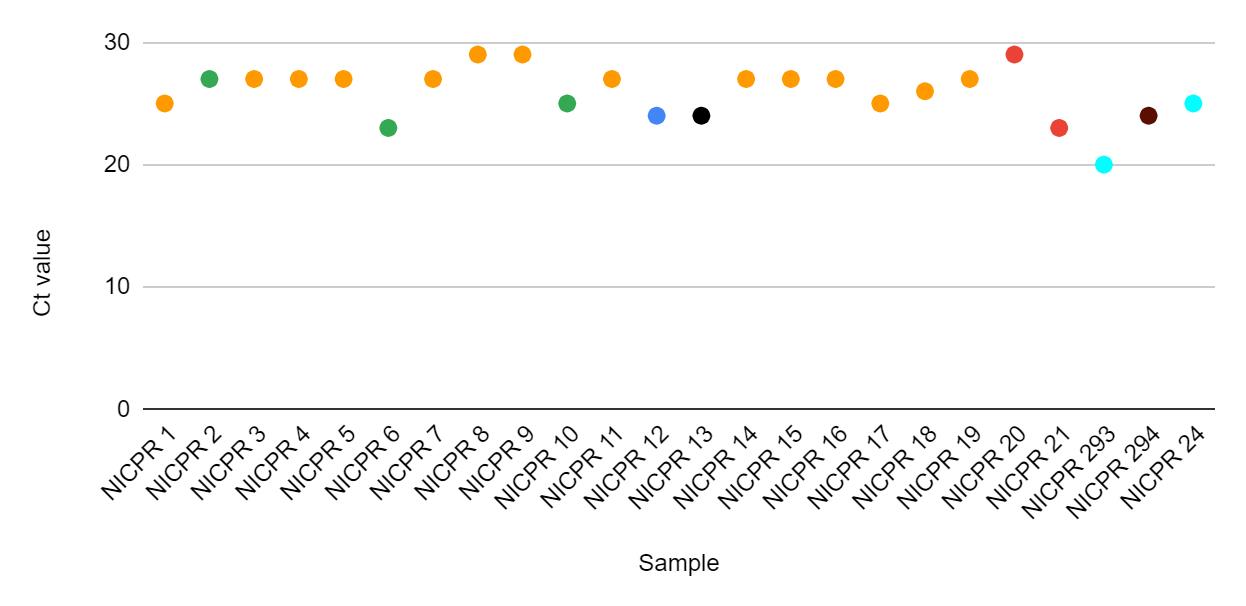
**

**Figure S1:** RT-qPCR based Ct values of ORF genes of the samples considered for whole genome sequencing.

**
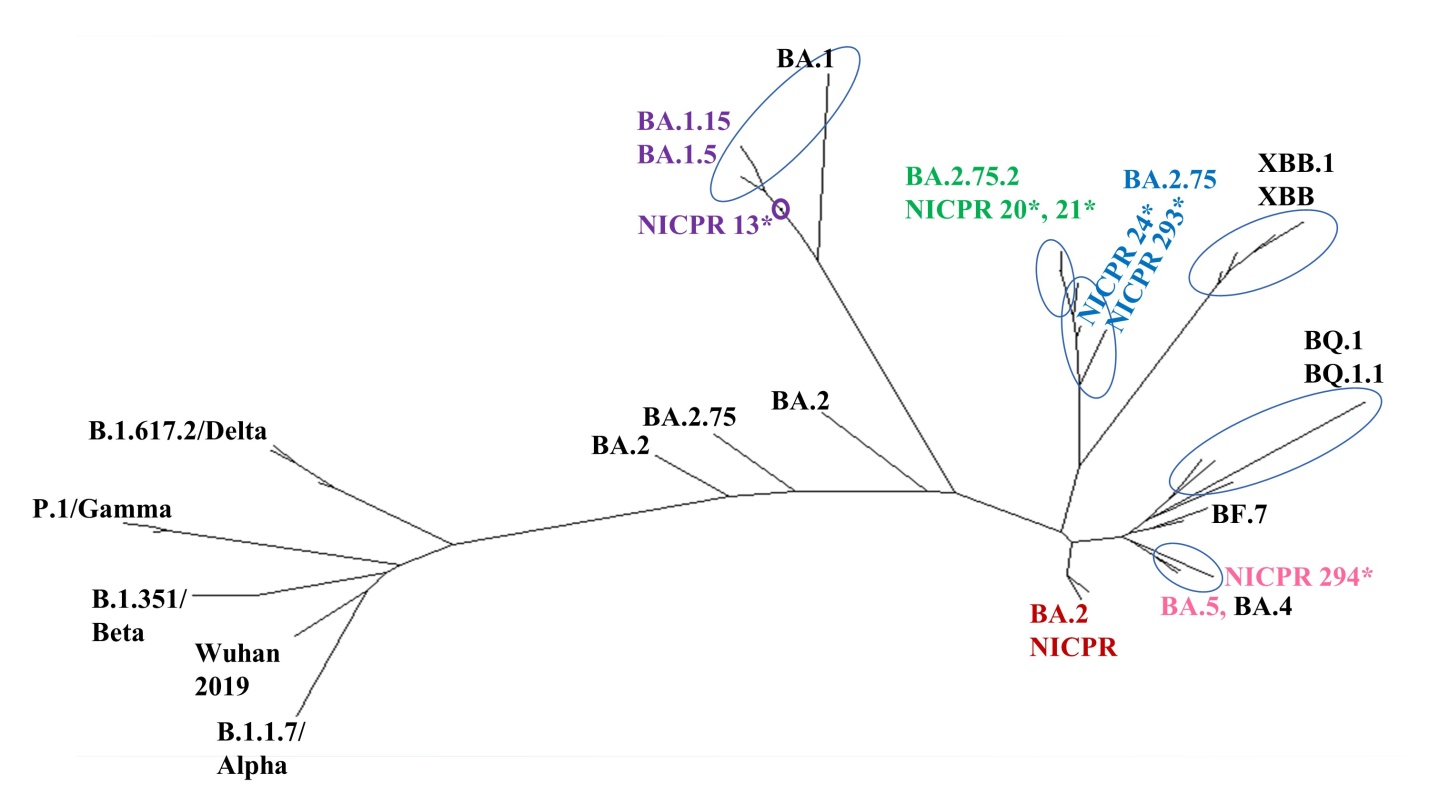
**

**Figure S2:** Phylogenetic relatedness of different SARS-CoV-2 genomes. Samples IDs with NICPR1 to NICPR24 belong to the current study.

**
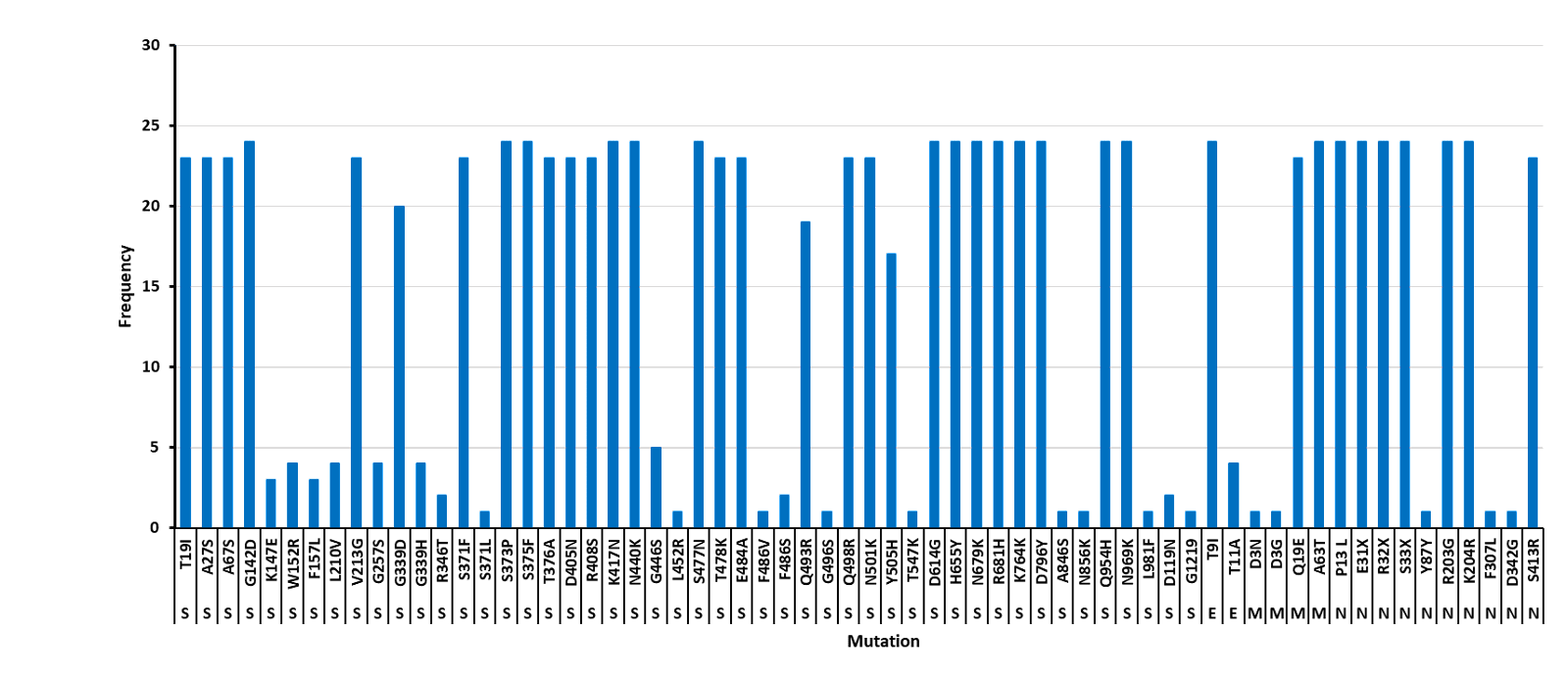
**

**Figure S3:** The frequency of amino acid mutations present in the structural proteins S, E, M, and N in all 24 viral genomes was mapped.

**Table TS2:** List of the samples submitted in the NCBI and GISAID with accession ID.

| **S. No.** | **Sample ID** | **NCBI Accession No.** | **GISAID Accession No.** |
| --- | --- | --- | --- |
|  | NICPR1 | OR357636 | EPI_ISL_17048977 |
|  | NICPR2 | OR357637 | EPI_ISL_17048978 |
|  | NICPR3 | OR357667 | EPI_ISL_17048979 |
|  | NICPR4 | OR357638 | EPI_ISL_17048980 |
|  | NICPR5 | OR357639 | EPI_ISL_17048981 |
|  | NICPR6 | OR357640 | EPI_ISL_16970178 |
|  | NICPR7 | OR357641 | EPI_ISL_16970179 |
|  | NICPR8 | OR357642 | EPI_ISL_17048982 |
|  | NICPR9 | OR357643 | EPI_ISL_16970180 |
|  | NICPR10 | OR357644 | EPI_ISL_16970181 |
|  | NICPR11 | OR357645 | EPI_ISL_17048983 |
|  | NICPR12 | OR357646 | EPI_ISL_17048984 |
|  | NICPR13 | OR357647 | EPI_ISL_17048985 |
|  | NICPR14 | OR357668 | EPI_ISL_17048986 |
|  | NICPR15 | OR357648 | EPI_ISL_16970182 |
|  | NICPR16 | OR357669 | EPI_ISL_16970183 |
|  | NICPR17 | OR357649 | EPI_ISL_17048987 |
|  | NICPR18 | OR357650 | EPI_ISL_17048988 |
|  | NICPR | OR394059 | EPI_ISL_17048989 |
|  | NICPR20 | OR357651 | EPI_ISL_17048990 |
|  | NICPR21 | OR357652 | EPI_ISL_17048991 |
|  | NICPR293 | OR357653 | EPI_ISL_16970184 |
|  | NICPR294 | OR357654 | EPI_ISL_16970185 |
|  | NICPR24 | OR357670 | EPI_ISL_17048992 |

**Table TS3: GISAID and Lineages details of the In-House samples:**

| **S.**  **No.** | **GISAID accession ID** | **Institute ID** | **Pango Lineage** | **Clade** | **Scorpio call** | **pangolin version** | **pangolin-data version** |
| --- | --- | --- | --- | --- | --- | --- | --- |
| 1 | EPI_ISL_17048977 | NICPR1 | BA.2 | GRA | Omicron (BA.2-like) | 4.2 | v1.18 |
| 2 | EPI_ISL_17048978 | NICPR2 | BA.2.10 | GRA | Omicron (BA.2-like) | 4.2 | v1.18 |
| 3 | EPI_ISL_17048979 | NICPR3 | BA.2 | GRA | Omicron (BA.2-like) | 4.2 | v1.18 |
| 4 | EPI_ISL_17048980 | NICPR4 | BA.2 | GRA | Omicron (BA.2-like) | 4.2 | v1.18 |
| 5 | EPI_ISL_17048981 | NICPR5 | BA.2 | GRA | Omicron (BA.2-like) | 4.2 | v1.18 |
| 6 | EPI_ISL_16970178 | NICPR6 | BA.2.10 | GRA | Omicron (BA.2-like) | 4.2 | v1.18 |
| 7 | EPI_ISL_16970179 | NICPR7 | BA.2 | GRA | Omicron (BA.2-like) | 4.2 | v1.18 |
| 8 | EPI_ISL_17048982 | NICPR8 | BA.2 | GRA | Omicron (BA.2-like) | 4.2 | v1.18 |
| 9 | EPI_ISL_16970180 | NICPR9 | BA.2 | GRA | Omicron (BA.2-like) | 4.2 | v1.18 |
| 10 | EPI_ISL_16970181 | NICPR10 | BA.2.10 | GRA | Omicron (BA.2-like) | 4.2 | v1.18 |
| 11 | EPI_ISL_17048983 | NICPR11 | BA.2 | GRA | Omicron (BA.2-like) | 4.2 | v1.18 |
| 12 | EPI_ISL_17048984 | NICPR12 | BA.2.37 | GRA | Omicron (BA.2-like) | 4.2 | v1.18 |
| 13 | EPI_ISL_17048985 | NICPR13 | BA.1.15 | GRA | Omicron (BA.1-like) | 4.2 | v1.18 |
| 14 | EPI_ISL_17048986 | NICPR14 | BA.2 | GRA | Omicron (BA.2-like) | 4.2 | v1.18 |
| 15 | EPI_ISL_16970182 | NICPR15 | BA.2 | GRA | Omicron (BA.2-like) | 4.2 | v1.18 |
| 16 | EPI_ISL_16970183 | NICPR16 | BA.2 | GRA | Omicron (BA.2-like) | 4.2 | v1.18 |
| 17 | EPI_ISL_17048987 | NICPR17 | BA.2 | GRA | Omicron (BA.2-like) | 4.2 | v1.18 |
| 18 | EPI_ISL_17048988 | NICPR18 | BA.2 | GRA | Omicron (BA.2-like) | 4.2 | v1.18 |
| 19 | EPI_ISL_17048989 | NICPR19 | BA.2 | GRA | Omicron (BA.2-like) | 4.2 | v1.18 |
| 20 | EPI_ISL_17048990 | NICPR20 | BA.2.75.2 | GRA | Omicron (BA.2-like) | 4.2 | v1.18 |
| 21 | EPI_ISL_17048991 | NICPR21 | BA.2.75.2 | GRA | Omicron (BA.2-like) | 4.2 | v1.18 |
| 22 | EPI_ISL_16970184 | NICPR293 | BA.2.75 | GRA | Omicron (BA.2-like) | 4.2 | v1.18 |
| 23 | EPI_ISL_16970185 | NICPR294 | BA.5.1 | GRA | Omicron (BA.5-like) | 4.2 | v1.18 |
| 24 | EPI_ISL_17048992 | NICPR24 | BA.2.75 | GRA | Omicron (BA.2-like) | 4.2 | v1.18 |

**Table TS4: Implications of mutations in spike protein**

| **S.No.** | **Wild residue** | **Position** | **Target residue** | **PredictSNP prediction** | **PredictSNP expected accuracy** | Annotations |
| --- | --- | --- | --- | --- | --- | --- |
| 1 | T | 19 | I | NEUTRAL | 0.65307311 |  |
| 2 | A | 27 | S | NEUTRAL | 0.82622462 |  |
| 3 | A | 67 | V | NEUTRAL | 0.73834499 | Natural variant: in strain: Eta/B.1.525, Omicron/BA.1 (mapped from position 67 in UniProt P0DTC2) |
| 4 | G | 142 | D | NEUTRAL | 0.74796037 | Natural variant: in strain: Kappa/B.1.617.1, Omicron/BA.2, Omicron/BA.2.12.1, Omicron/BA.4, Omicron/BA.5 (mapped from position 142 in UniProt P0DTC2) |
| 5 | K | 147 | E | NEUTRAL | 0.73834499 |  |
| 6 | **W** | **152** | **R** | **DELETERIOUS** | **0.60548272** |  |
| 7 | F | 157 | L | NEUTRAL | 0.73688811 | Natural variant: in strain: A23.1 (mapped from position 157 in UniProt P0DTC2) |
| 8 | I | 210 | V | NEUTRAL | 0.82622462 |  |
| 9 | V | 213 | G | NEUTRAL | 0.62831343 | Natural variant: in strain: Omicron/BA.2, Omicron/BA.2.12.1, Omicron/BA.4, Omicron/BA.5 (mapped from position 213 in UniProt P0DTC2) |
| 10 | G | 257 | S | NEUTRAL | 0.82622462 |  |
| 11 | **G** | **339** | **H** | **DELETERIOUS** | **0.63587604** |  |
| 12 | G | 339 | D | NEUTRAL | 0.73834499 | Natural variant: in strain: Omicron/BA.1, Omicron/BA.2, Omicron/BA.2.12.1, Omicron/BA.4, Omicron/BA.5 (mapped from position 339 in UniProt P0DTC2) |
| 13 | R | 346 | T | NEUTRAL | 0.65307311 |  |
| 14 | S | 371 | F | NEUTRAL | 0.73834499 |  |
| 15 | S | 373 | P | NEUTRAL | 0.82622462 | Natural variant: in strain: Omicron/BA.1, Omicron/BA.2, Omicron/BA.2.12.1, Omicron/BA.4, Omicron/BA.5 (mapped from position 373 in UniProt P0DTC2) |
| 16 | S | 375 | F | NEUTRAL | 0.75203963 | Natural variant: in strain: Omicron/BA.1, Omicron/BA.2, Omicron/BA.2.12.1, Omicron/BA.4, Omicron/BA.5 (mapped from position 375 in UniProt P0DTC2) |
| 17 | T | 376 | A | NEUTRAL | 0.65307311 | Natural variant: in strain: Omicron/BA.2, Omicron/BA.2.12.1, Omicron/BA.4, Omicron/BA.5 (mapped from position 376 in UniProt P0DTC2) |
| 18 | D | 405 | N | NEUTRAL | 0.62529138 | Natural variant: in strain: Omicron/BA.2, Omicron/BA.2.12.1, Omicron/BA.4, Omicron/BA.5 (mapped from position 405 in UniProt P0DTC2) |
| 19 | R | 408 | S | NEUTRAL | 0.73688811 | Natural variant: in strain: Omicron/BA.2, Omicron/BA.2.12.1, Omicron/BA.4, Omicron/BA.5 (mapped from position 408 in UniProt P0DTC2) |
| 20 | K | 417 | N | NEUTRAL | 0.73834499 | Natural variant: in strain: Beta/B.1.351, Gamma/P.1, Omicron/BA.1, Omicron/BA.2, Omicron/BA.2.12.1, Omicron/BA.4, Omicron/BA.5; May enhance affinity to human ACE2 receptor (mapped from position 417 in UniProt P0DTC2) |
| 21 | N | 440 | K | NEUTRAL | 0.73688811 | Natural variant: in strain: Omicron/BA.1, Omicron/BA.2, Omicron/BA.2.12.1, Omicron/BA.4, Omicron/BA.5 (mapped from position 440 in UniProt P0DTC2) |
| 22 | G | 446 | S | NEUTRAL | 0.82622462 | Natural variant: in strain: Omicron/BA.1 (mapped from position 446 in UniProt P0DTC2) |
| 23 | **L** | **452** | **R** | **DELETERIOUS** | **0.65494636** | **Natural variant: in strain: Delta/B.1.617.2, Epsilon/B.1.427/B.1.429, Kappa/B.1.617.1, Lambda/C.37, 19B/501Y, Omicron/BA.4, Omicron/BA.5; Contributes to cellular immunity evasion and increases infectivity (mapped from position 452 in UniProt P0DTC2)** |
| 24 | N | 460 | K | NEUTRAL | 0.82622462 |  |
| 25 | S | 477 | N | NEUTRAL | 0.82622462 | Natural variant: in strain: 20A.EU2, Iota/B.1.526, Omicron/BA.1, Omicron/BA.2, Omicron/BA.2.12.1, Omicron/BA.4, Omicron/BA.5 (mapped from position 477 in UniProt P0DTC2) |
| 26 | T | 478 | K | NEUTRAL | 0.63151762 | Natural variant: in strain: Delta/B.1.617.2, Omicron/BA.1, Omicron/BA.2, Omicron/BA.2.12.1, Omicron/BA.4, Omicron/BA.5 (mapped from position 478 in UniProt P0DTC2) |
| 27 | E | 484 | A | NEUTRAL | 0.82622462 | Natural variant: in strain: Omicron/BA.1, Omicron/BA.2 Omicron/BA.2.12.1, Omicron/BA.4, Omicron/BA.5 (mapped from position 484 in UniProt P0DTC2) |
| 28 | F | 486 | V | NEUTRAL | 0.68365861 | Natural variant: in strain: Omicron/BA.4, Omicron/BA.5 (mapped from position 486 in UniProt P0DTC2) |
| 29 | F | 486 | S | NEUTRAL | 0.82622462 |  |
| 30 | Q | 493 | R | NEUTRAL | 0.75203963 | Natural variant: in strain: Omicron/BA.1, Omicron/BA.2 Omicron/BA.2.12.1 (mapped from position 493 in UniProt P0DTC2) |
| 31 | G | 496 | S | NEUTRAL | 0.63151762 | Natural variant: in strain: Omicron/BA.1 (mapped from position 496 in UniProt P0DTC2) |
| 32 | Q | 498 | R | NEUTRAL | 0.68365861 | Natural variant: in strain: Omicron/BA.1, Omicron/BA.2, Omicron/BA.2.12.1, Omicron/BA.4, Omicron/BA.5 (mapped from position 498 in UniProt P0DTC2) |
| 33 | N | 501 | Y | NEUTRAL | 0.6025641 | Natural variant: in strain: Alpha/B.1.1.7, Beta/B.1.351, Gamma/P.1, Theta/P.3, Mu/B.1.621, 19B/501Y, Omicron/BA.1, Omicron/BA.2, Omicron/BA.2.12.1, Omicron/BA.4, Omicron/BA.5; May enhance affinity to human ACE2 receptor (mapped from position 501 in UniProt P0DTC2) |
| 34 | **Y** | **505** | **H** | **DELETERIOUS** | **0.60697259** | **Natural variant: in strain: Omicron/BA.1, Omicron/BA.2, Omicron/BA.2.12.1, Omicron/BA.4, Omicron/BA.5 (mapped from position 505 in UniProt P0DTC2)** |
| 35 | T | 547 | K | NEUTRAL | 0.82622462 | Natural variant: in strain: Omicron/BA.1 (mapped from position 547 in UniProt P0DTC2) |
| 36 | D | 614 | G | NEUTRAL | 0.82622462 | Natural variant: in strain: Alpha/B.1.1.7, Beta/B.1.351, Gamma/P.1, Delta/B.1.617.2, Epsilon/B.1.427/B.1.429, Eta/B.1.525, Theta/P.3, Iota/B.1.526, Kappa/B.1.617.1, Lambda/C.37, B.1.1.318, Zeta/P.2, Mu/B.1.621, 20A.EU1, 20A.EU2, Omicron/BA.1, Omicron/BA.2, Omicron/BA.2.12.1, Omicron/BA.4, Omicron/BA.5; common variant; binds to hACE2 more efficiently; produces more infectious particles when cultured in primary human epithelial cells; does not significantly shift SARS-CoV-2 neutralization properties (mapped from position 614 in UniProt P0DTC2) |
| 37 | H | 655 | Y | NEUTRAL | 0.73688811 | Natural variant: in strain: Gamma/P.1, 19B/501T, 19B/501Y, Omicron/BA.1, Omicron/BA.2, Omicron/BA.2.12.1, Omicron/BA.4, Omicron/BA.5 (mapped from position 655 in UniProt P0DTC2) |
| 38 | N | 679 | K | NEUTRAL | 0.82622462 | Natural variant: in strain: Omicron/BA.1, Omicron/BA.2, Omicron/BA.2.12.1, Omicron/BA.4, Omicron/BA.5 (mapped from position 679 in UniProt P0DTC2) |
| 39 | P | 681 | H | NEUTRAL | 0.75291375 | Natural variant: in strain: Alpha/B.1.1.7, Theta/P.3, Mu/B.1.621, B.1.1.318, A23.1, Omicron/BA.1, Omicron/BA.2, Omicron/BA.2.12.1, Omicron/BA.4, Omicron/BA.5 (mapped from position 681 in UniProt P0DTC2) |
| 40 | **N** | **764** | **K** | **DELETERIOUS** | **0.71871275** | **Natural variant: in strain: Omicron/BA.1, Omicron/BA.2, Omicron/BA.2.12.1, Omicron/BA.4, Omicron/BA.5 (mapped from position 764 in UniProt P0DTC2)** |
| 41 | D | 796 | Y | NEUTRAL | 0.75203963 | Natural variant: in strain: 19B/501Y, Omicron/BA.1, Omicron/BA.2, Omicron/BA.2.12.1, Omicron/BA.4, Omicron/BA.5 (mapped from position 796 in UniProt P0DTC2) |
| 42 | A | 846 | S | NEUTRAL | 0.65307311 |  |
| 43 | **N** | **856** | **K** | **DELETERIOUS** | **0.7556615** | **Natural variant: in strain: Omicron/BA.1 (mapped from position 856 in UniProt P0DTC2)** |
| 44 | Q | 954 | H | NEUTRAL | 0.73688811 | Natural variant: in strain: Omicron/BA.1, Omicron/BA.2, Omicron/BA.2.12.1, Omicron/BA.4, Omicron/BA.5 (mapped from position 954 in UniProt P0DTC2) |
| 45 | **N** | **969** | **K** | **DELETERIOUS** | **0.54946365** | **Natural variant: in strain: Omicron/BA.1, Omicron/BA.2, Omicron/BA.2.12.1, Omicron/BA.4, Omicron/BA.5 (mapped from position 969 in UniProt P0DTC2)** |
| 46 | L | 981 | F | NEUTRAL | 0.82622462 | Natural variant: in strain: Omicron/BA.1 (mapped from position 981 in UniProt P0DTC2) |
| 47 | D | 1199 | N | NEUTRAL | 0.65307311 |  |
| 48 | G | 1219 | V | NEUTRAL | 0.6025641 | Natural variant: in strain: 19B/501Y (mapped from position 1219 in UniProt P0DTC2) |

**Table TS5: N- Glycosylation sites in In-house sample**

**
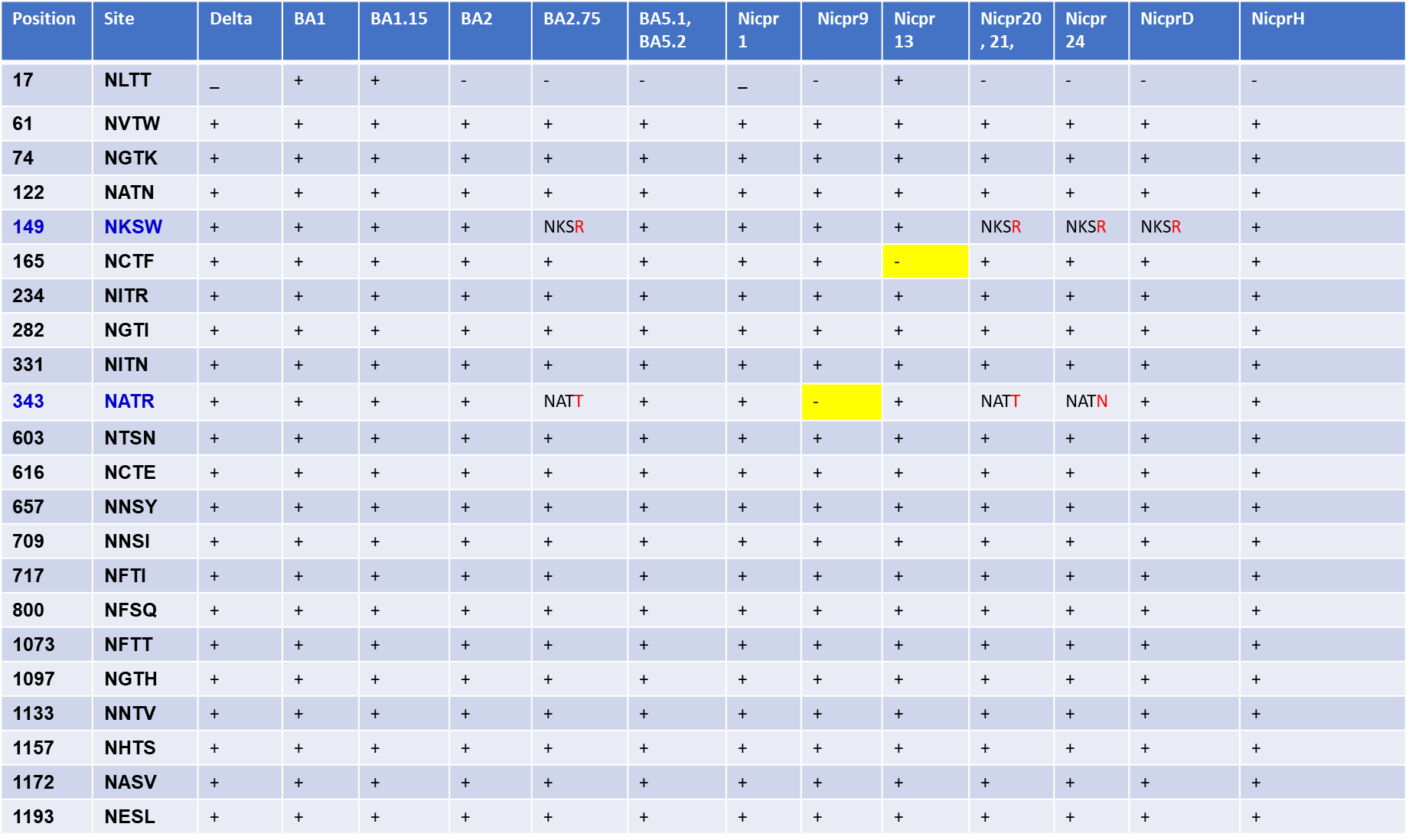
**

**Table TS6: Predicted N glycosylation sites with their estimated values**

| **Samples** | **Position & Sequence** | **Value** |
| --- | --- | --- |
| **WT SARS-CoV-2** | **149 NKSW** | **0.6318 (7/9) +** |
| **BA.2.75.2** | **145 NKSR** | **0.6946 (8/9) +** |
| **NICPR20** | **145 NKSR** | **0.6947 (8/9) +** |
| **NICPR21** | **145 NKSR** | **0.6946 (8/9) +** |
| **NICPR24** | **145 NKSR** | **0.6948 (8/9) +** |
| **NICPR293D** | **142 NKSR** | **0.6744 (9/9) ++** |
| **NICPR1** | **145 NKSW** | **0.6348 (7/9) +** |
| **NICPR2** | **145 NKSW** | **0.6348 (7/9) +** |
| **NICPR3** | **145 NKSW** | **0.6348 (7/9) +** |
| **NICPR4** | **145 NKSW** | **0.6348 (7/9) +** |
| **NICPR5** | **145 NKSW** | **0.6348 (7/9) +** |
| **NICPR6** | **145 NKSW** | **0.6348 (7/9) +** |
| **NICPR7** | **145 NKSW** | **0.6348 (7/9) +** |
| **NICPR9** | **145 NKSW** | **0.6348 (7/9) +** |
| **NICPR10** | **145 NKSW** | **0.6348 (7/9) +** |
| **NICPR11** | **144 NKSW** | **0.6348 (7/9) +** |
| **NICPR12** | **144 NKSW** | **0.6348 (7/9) +** |
| **NICPR13** | **141 NKSW** | **0.5890 (6/9) +** |
| **NICPR14** | **145 NKSW** | **0.6348 (7/9) +** |
| **NICPR15** | **145 NKSW** | **0.6348 (7/9) +** |
| **NICPR16** | **144 NKSW** | **0.6348 (7/9) +** |
| **NICPR17** | **145 NKSW** | **0.6348 (7/9) +** |
| **NICPR18** | **144 NKSW** | **0.6348 (7/9) +** |
| **NICPR19** | **144 NKSW** | **0.6348 (7/9) +** |
| **NICPR294H** | **141 NKSW** | **0.6351 (7/9) +** |
| **At Position 343** | | |
| **WT SARS-CoV-2** | **343 NATR** | **0.5671 (8/9) +** |
| **BA.2.75.2** | **339 NATT** | **0.5497 (6/9) +** |
| **NICPR20** | **339 NATT** | **0.5497 (6/9) +** |
| **NICPR21** | **339 NATT** | **0.5497 (6/9) +** |
| **NICPR24** | **339 NATN** | **0.5751 (7/9) +** |
| **NICPR293D** | **336 NATR** | **0.6216 (9/9) ++** |
| **NICPR294H** | **335 NATR** | **0.5714 (8/9) +** |
| **NICPR1** | **339 NATR** | **0.5704 (8/9) +** |
| **NICPR2** | **339 NATR** | **0.5704 (8/9) +** |
| **NICPR3** | **339 NATR** | **0.5704 (8/9) +** |
| **NICPR4** | **339 NATR** | **0.5704 (8/9) +** |
| **NICPR5** | **339 NATR** | **0.5704 (8/9) +** |
| **NICPR6** | **339 NATR** | **0.5704 (8/9) +** |
| **NICPR7** | **339 NATR** | **0.5704 (8/9) +** |
| **NICPR10** | **339 NATR** | **0.5704(8/9) +** |
| **NICPR11** | **338 NATR** | **0.5706 (8/9) +** |
| **NICPR12** | **337 NATR** | **0.5744 (6/9) +** |
| **NICPR13** | **327 NATR** | **0.5721 (8/9) +** |
| **NICPR14** | **339 NATR** | **0.5704 (8/9) +** |
| **NICPR15** | **339 NATR** | **0.5704 (8/9) +** |
| **NICPR16** | **338 NATR** | **0.5706 (8/9) +** |
| **NICPR17** | **339 NATR** | **0.5704 (8/9) +** |
| **NICPR18** | **338 NATR** | **0.5706 (8/9) +** |
| **NICPR19** | **337 NATR** | **0.5706 (8/9) +** |

**Table TS7: Predicted helper T cell epitopes**

| **S No** | **Sequence** | **Samples** | **Alleles** |
| --- | --- | --- | --- |
| 1 | FNGL**T**GTGV/  FNGL**K**GTGV | WT SARS-CoV-2, NICPR13, NICPR20, NICPR21, NICPR293 & NICPR294/ NICPR13 | DRB1_0101 |
| 2 | FELLHAPAT | WT SARS-CoV-2, NICPR13, NICPR20, NICPR21, NICPR293 & NICPR294 | DRB1_0101 |
| 3 | FRVQPTESI | WT SARS-CoV-2, NICPR13, NICPR20, NICPR21, NICPR293 & NICPR294 | DRB1_0101, DRB1_1302 |
| 4 | **FHAISGTNG/FHVISGTNG** | NICPR293/ NICPR13 | DRB1_0101 |
| 5 | FVTQRNFYE | WT SARS-CoV-2, NICPR13, NICPR20, NICPR21, NICPR293 & NICPR294 | DRB1_0405 |
| 6 | LTGIAVEQD | WT SARS-CoV-2, NICPR13, NICPR20, NICPR21, NICPR293 & NICPR294 | DRB1_0405,  HLA-DQA10401-DQB10401  HLA-DQA10102-DQB10202,  HLA-DQA10402-DQB10402 |
| 7 | FSALEPLVD | WT SARS-CoV-2, NICPR13, NICPR20, NICPR21, NICPR293 & NICPR294 | DRB1_0405 |
| 8 | YFKNHTSPD | WT SARS-CoV-2, NICPR13, NICPR20, NICPR21, NICPR293 & NICPR294 | DRB1_0405 |
| 9 | YDPLQPELD | WT SARS-CoV-2, NICPR13, NICPR20, NICPR21, NICPR293 & NICPR294 | DRB1_0405 |
| 10 | IYQTSNFRV | WT SARS-CoV-2, NICPR13, NICPR20, NICPR21, NICPR293 & NICPR294 | DRB1_0701, DRB1_1302, HLA-DQA10102-DQB10202, HLA-DQA10101-DQB10201, HLA-DQA10501-DQB10501 |
| 11 | FTISVTTEI | WT SARS-CoV-2, NICPR13, NICPR20, NICPR21, NICPR293 & NICPR294 | DRB1_0701 |
| 12 | FQPTNGVGY | WT SARS-CoV-2 | DRB1_0701 |
| 13 | VYSTGSNVF | WT SARS-CoV-2, NICPR13, NICPR20, NICPR21, NICPR293 & NICPR294 | DRB1_0701 |
| 14 | IAYTMSLGA | WT SARS-CoV-2, NICPR13, NICPR20, NICPR21, NICPR293 & NICPR294 | DRB1_0701 |
| 15 | FKNHTSPDV | WT SARS-CoV-2, NICPR13, NICPR20, NICPR21, NICPR293 & NICPR294 | DRB1_0701 |
| 16 | YTNSFTRGV | WT SARS-CoV-2, NICPR13, NICPR20, NICPR21, NICPR293 & NICPR294 & NICPR13 | DRB1_0701 |
| 17 | **YSKHTPIIV** | NICPR13 | DRB1_0701 |
| 18 | **FVIRGNEVS** | NICPR20, NICPR21, NICPR293, NICPR294 | DRB1_0802, DRB1_1302 |
| 19 | **FSRLDKVEA** | NICPR13 | DRB1_0802 |
| 20 | FKNLREFVF | WT SARS-CoV-2, NICPR13, NICPR20, NICPR21, NICPR293 & NICPR294 | DRB1_1201 |
| 21 | IGIVNNTVY | WT SARS-CoV-2, NICPR13, NICPR20, NICPR21, NICPR293 & NICPR294 | DRB1_1201 |
| 22 | VVFLHVTYV | WT SARS-CoV-2, NICPR13, NICPR20, NICPR21, NICPR293 & NICPR294 | DRB1_1201 |
| 23 | IGVTQNVLY | WT SARS-CoV-2, NICPR13, NICPR20, NICPR21, NICPR293 & NICPR294 | DRB1_1201 |
| 24 | VFAQVKQIY | WT SARS-CoV-2, NICPR13, NICPR20, NICPR21, NICPR293 & NICPR294 | DRB1_1201 |
| 25 | IYKTPPIKY | NICPR13, NICPR20, NICPR21, NICPR293 & NICPR294 | DRB1_1201, DRB1_1302 |
| 26 | LIVNNATNV | WT SARS-CoV-2, NICPR13, NICPR20, NICPR21, NICPR293 & NICPR294 | DRB1_1302 |
| 27 | IVNNATNVV | WT SARS-CoV-2, NICPR13, NICPR20, NICPR21, NICPR293 & NICPR294 | DRB1_1302 |
| 28 | IRASANLAA | WT SARS-CoV-2, NICPR13, NICPR20, NICPR21, NICPR293 & NICPR294 | DRB1_1302, HLA-DQA10101-DQB10201, HLA-DQA10102-DQB10202, HLA-DQA10501-DQB10501 |
| 29 | FVSNGTHWF | WT SARS-CoV-2, NICPR13, NICPR20, NICPR21, NICPR293 & NICPR294 | DRB1_1302 |
| 30 | LYENQKLIA | WT SARS-CoV-2, NICPR13, NICPR20, NICPR21, NICPR293 & NICPR294 | DRB1_1302 |
| 31 | LLFNKVTLA | WT SARS-CoV-2, NICPR13, NICPR20, NICPR21, NICPR293 & NICPR294 | DRB1_1302 |
| 32 | LKYNENGTI | WT SARS-CoV-2, NICPR13, NICPR20, NICPR21, NICPR293 & NICPR294 | DRB1_1302 |
| 33 | ILPDPSKPS | WT SARS-CoV-2, NICPR13, NICPR20, NICPR21, NICPR293 & NICPR294 | DRB1_1302, HLA-DQA10101-DQB10201, HLA-DQA10102-DQB10202 |
| 34 | FRVQPTESI | WT SARS-CoV-2, NICPR13, NICPR20, NICPR21, NICPR293 & NICPR294 | DRB1_1302 |
| 35 | IITTDNTFV | WT SARS-CoV-2, NICPR13, NICPR20, NICPR21, NICPR293 & NICPR294 | DRB1_1302 |
| 36 | IAWNSNNLD | WT SARS-CoV-2 | DRB1_1302 |
| 37 | **YHKNNKSRM** | NICPR293 | DRB1_1302 |
| 38 | **IRGNEVSQI** | NICPR20, NICPR21, NICPR293 & NICPR294 | DRB1_1302 |
| 39 | VIAWNSNKL | NICPR13, NICPR20, NICPR21, NICPR293 & NICPR294 | DRB1_1302, HLA-DQA10501-DQB10501 |
| 40 | VVNHNAQAL | NICPR13, NICPR20, NICPR21, NICPR293 & NICPR294 | DRB1_1302 |
| 41 | FNATRFASV | WT SARS-CoV-2, NICPR13, NICPR293, NICPR294 | HLA-DPA10103-DPB110101, HLA-DPA10201-DPB110201, HLA-DPA10301-DPB110301 |
| 42 | FLGVYYHKN | WT SARS-CoV-2 | HLA-DPA10103-DPB110101, HLA-DPA10301-DPB110301 |
| 43 | LIANQFNSA | WT SARS-CoV-2, NICPR13, NICPR20, NICPR21, NICPR293 & NICPR294 | HLA-DPA10103-DPB110101, HLA-DPA10201-DPB110201, HLA-DPA10301-DPB110301 |
| 44 | YLYRLFRKS | WT SARS-CoV-2, NICPR13, NICPR20, NICPR21, NICPR293, | HLA-DPA10103-DPB110101, HLA-DPA10201-DPB110201 |
| 45 | INITRFQTL | WT SARS-CoV-2, NICPR13, NICPR20, NICPR21, NICPR293 & NICPR294 | HLA-DPA10103-DPB110101, HLA-DPA10301-DPB110301 |
| 46 | YQTSNFRVQ | WT SARS-CoV-2, NICPR13, NICPR20, NICPR21, NICPR293 & NICPR294 | HLA-DPA10103-DPB110101, HLA-DPA10201-DPB110201, HLA-DPA10301-DPB110301 |
| 47 | FRSSVLHST | WT SARS-CoV-2, NICPR13, NICPR20, NICPR21, NICPR293 & NICPR294 | HLA-DPA10103-DPB110101, HLA-DPA10201-DPB110201, HLA-DPA10301-DPB110301, HLA-DQA10301-DQB10301 |
| 48 | FLHVTYVPA | WT SARS-CoV-2, NICPR13, NICPR20, NICPR21, NICPR293 & NICPR294 | HLA-DPA10103-DPB110101, HLA-DPA10201-DPB110201, HLA-DPA10301-DPB110301 |
| 49 | FGGFNFSQI | WT SARS-CoV-2, NICPR13, NICPR20, NICPR21, NICPR293 & NICPR294 | HLA-DPA10103-DPB110101, HLA-DPA10301-DPB110301 |
| 50 | LREFVFKNI | WT SARS-CoV-2, NICPR13, NICPR20, NICPR21, NICPR293 & NICPR294 | HLA-DPA10103-DPB110101, HLA-DPA10301-DPB110301 |
| 51 | VGYQPYRVV | WT SARS-CoV-2, NICPR20 | HLA-DPA10103-DPB110101 |
| 52 | LQSYGFQPT | WT SARS-CoV-2 | HLA-DPA10103-DPB110101 |
| 53 | YVSQPFLMD | WT SARS-CoV-2, NICPR20, NICPR21, NICPR293 & NICPR294 | HLA-DPA10103-DPB110101, HLA-DPA10201-DPB110201 |
| 54 | IEDLLFNKV | WT SARS-CoV-2, NICPR20, NICPR21, NICPR293 & NICPR294 | HLA-DPA10103-DPB110101, HLA-DPA10301-DPB110301 |
| 55 | **FLDVYYHEN/**  **FLDVYYHKN** | NICPR20, NICPR21/ NICPR293, NICPR294 | HLA-DPA10103-DPB110101, HLA-DPA10201-DPB110201, HLA-DQA10301-DQB10301/ HLA-DPA10103-DPB110101, HLA-DPA10201-DPB110201 |
| 56 | ISTEIYQAG | WT SARS-CoV-2, NICPR13, NICPR20, NICPR21, NICPR293 & NICPR294 | HLA-DPA10201-DPB110201 |
| 57 | KKFLPFQQF | WT SARS-CoV-2, NICPR13, NICPR20, NICPR21, NICPR293 & NICPR294 | HLA-DPA10201-DPB110201, HLA-DPA10301-DPB110301 |
| 58 | FNFSQILPD | WT SARS-CoV-2, NICPR13, NICPR20, NICPR21, NICPR293 & NICPR294 | HLA-DPA10201-DPB110201, HLA-DQA10401-DQB10401, HLA-DQA10501-DQB10501, HLA-DQA10402-DQB10402 |
| 59 | INLVRDLPQ | WT SARS-CoV-2 | HLA-DQA10101-DQB10201, HLA-DQA10501-DQB10501, HLA-DQA10102-DQB10202 |
| 60 | IADTTDAVR | WT SARS-CoV-2, NICPR13, NICPR20, NICPR21, NICPR293 & NICPR294 | HLA-DQA10101-DQB10201, HLA-DQA10102-DQB10202 |
| 61 | FASVYAWNR | WT SARS-CoV-2, NICPR13, NICPR20, NICPR21 | HLA-DQA10101-DQB10201 |
| 62 | ITTDNTFVS | WT SARS-CoV-2, NICPR13, NICPR20, NICPR21, NICPR293 & NICPR294 | HLA-DQA10101-DQB10201 |
| 63 | IRAAEIRAS | WT SARS-CoV-2, NICPR13, NICPR20, NICPR21, NICPR293 & NICPR294 | HLA-DQA10301-DQB10301, HLA-DQA10401-DQB10401, HLA-DQA10501-DQB10501, HLA-DQA10102-DQB10202, HLA-DQA10402-DQB10402 |
| 64 | VYFASTEKS | WT SARS-CoV-2, NICPR13, NICPR20, NICPR21, NICPR293 & NICPR294 | HLA-DQA10301-DQB10301, HLA-DQA10402-DQB10402, HLA-DQA10401-DQB10401 |
| 65 | WTAGAAAYY | WT SARS-CoV-2, NICPR13, NICPR20, NICPR21, NICPR293 & NICPR294 | HLA-DQA10301-DQB10301, HLA-DQA10401-DQB10401, HLA-DQA10402-DQB10402 |
| 66 | INASVVNIQ | WT SARS-CoV-2, NICPR13, NICPR20, NICPR21, NICPR293 & NICPR294 | HLA-DQA10301-DQB10301, HLA-DQA10401-DQB10401, HLA-DQA10402-DQB10402 |
| 67 | FTGCVIAWN | WT SARS-CoV-2, NICPR13, NICPR20, NICPR21, NICPR293 & NICPR294 | HLA-DQA10301-DQB10301 |
| 68 | TAGAAAYYV | WT SARS-CoV-2, NICPR13, NICPR20, NICPR21, NICPR293 & NICPR294 | HLA-DQA10401-DQB10401, HLA-DQA10402-DQB10402 |
| 69 | ADTTDAVRD | WT SARS-CoV-2, NICPR13, NICPR20, NICPR21, NICPR293 & NICPR294 | HLA-DQA10401-DQB10401, HLA-DQA10402-DQB10402 |
| 70 | VLPFNDGVY | WT SARS-CoV-2, NICPR13, NICPR20, NICPR21, NICPR293 & NICPR294 | HLA-DQA10501-DQB10501, |
| 71 | IAQYTSALL | WT SARS-CoV-2, NICPR13, NICPR20, NICPR21, NICPR293 & NICPR294 | HLA-DQA10501-DQB10501 |
| 72 | VIAWNSNNL | WT SARS-CoV-2 | HLA-DQA10501-DQB10501, HLA-DQA10102-DQB10202 |
| 73 | IRGDEVRQI | NICPR13 & WT SARS-CoV-2 | HLA-DQA10102-DQB10202 |

**Table TS8: All predicted cytotoxic T cell epitopes**

| **SN** | **Position** | **Sequence** | **Samples** | **Allele** |
| --- | --- | --- | --- | --- |
| **1** | **612** | **VLYQDVNCTEV/**  **VLYQGVNCTEV** | **WT SARS-CoV-2/WT SARS-CoV-2, NICPR13, NICPR20, NICPR21, NICPR293 & NICPR294** | **HLA-A02:01** |
| **2** | **132** | **FQFCNDPFLGV** | **WT SARS-CoV-2** | **HLA-A02:01** |
| **3** | **321** | **NLCPFDEVFNA** | **NICPR13 & NICPR294** | **HLA-A02:01** |
| **4** | **516** | **SFELLHAPATV** | **WT SARS-CoV-2, NICPR13, NICPR20, NICPR21, NICPR293 & NICPR294** | **HLA-A02:01** |
| **5** | **985** | **RLDKVEAEVQI** | **WT SARS-CoV-2, NICPR13, NICPR20, NICPR21, NICPR293 & NICPR294** | **HLA-A02:01** |
| **6** | **80** | **VLPFNDGVYFA** | **WT SARS-CoV-2, NICPR13, NICPR20, NICPR21, NICPR293 & NICPR294** | **HLA-A02:01** |
| **7** | **976** | **SSVLNDILSRL** | **WT SARS-CoV-2, NICPR20, NICPR21, NICPR293 & NICPR294** | **HLA-A02:01** |
| **8** | **5** | **VLLPLVSSQCV** | **WT SARS-CoV-2, NICPR13, NICPR20, NICPR21, NICPR293 & NICPR294** | **HLA-A02:01** |
| **9** | **1048** | **GYHLMSFPQSA** | **WT SARS-CoV-2, NICPR13, NICPR20, NICPR21, NICPR293 & NICPR294** | **HLA-A02:01** |
| **10** |  | **FSRLDKVEAEV (968)** | **NICPR13 only** | **HLA-A02:01** |
| **11** |  | **AKNLNESLINL (1086)** | **NICPR20, NICPR21** | **HLA-A02:01** |
| **12** | **725** | **TTLPVSMTK** | **WT SARS-CoV-2, NICPR13, NICPR20, NICPR21, NICPR293 & NICPR294** | **HLA-A11:01** |
| **13** | **827** | **KVADAGFIK** | **WT SARS-CoV-2, NICPR13, NICPR20, NICPR21, NICPR293 & NICPR294** | **HLA-A11:01** |
| **14** | **817** | **RSFDLLFNK** | **WT SARS-CoV-2, NICPR13, NICPR20, NICPR21, NICPR293 & NICPR294** | **HLA-A11:01** |
| **15** | **1078** | **TTAPAICHK** | **WT SARS-CoV-2, NICPR13, NICPR20, NICPR21, NICPR293 & NICPR294** | **HLA-A11:01** |
| **16** | **913** | **VTQNVLNQK** | **WT SARS-CoV-2, NICPR13, NICPR20, NICPR21, NICPR293 & NICPR294** | **HLA-A11:01** |
| **17** | **1173** | **GINASVIQK** | **WT SARS-CoV-2, NICPR13, NICPR20, NICPR21, NICPR293 & NICPR294** | **HLA-A11:01** |
| **18** | **806** | **QILPDPPSK** | **WT SARS-CoV-2, NICPR13, NICPR20, NICPR21, NICPR293 & NICPR294** | **HLA-A11:01** |
| **19** | **550** | **GTLTESNKK** | **WT SARS-CoV-2, NICPR13, NICPR20, NICPR21, NICPR293 & NICPR294** | **HLA-A11:01** |
| **20** | **674** | **ASYQTQTPR** | **WT SARS-CoV-2** | **HLA-A11:01** |
| **21** | **118** | **IVNNATVIK** | **WT SARS-CoV-2, NICPR13, NICPR20, NICPR21, NICPR293 & NICPR294** | **HLA-A11:01** |
| **22** | **406** | **VIAPGQTGK** | **WT SARS-CoV-2** | **HLA-A11:01** |
| **23** | **782** | **EVFAKQIYK** | **WT SARS-CoV-2, NICPR13, NICPR20, NICPR21, NICPR293 & NICPR294** | **HLA-A11:01** |
| **24** | **1065** | **LTYVPAQEK** | **WT SARS-CoV-2, NICPR13, NICPR20, NICPR21, NICPR293 & NICPR294** | **HLA-A11:01** |
| **25** | **939** | **SSTASALGK** | **WT SARS-CoV-2, NICPR13, NICPR20, NICPR21, NICPR293 & NICPR294** | **HLA-A11:01** |
| **26** | **191** | **FVFKDGYFK** | **WT SARS-CoV-2, NICPR13, NICPR20, NICPR21, NICPR293 & NICPR294** | **HLA-A11:01** |
| **27** | **413** | **QTIADYNYK** | **WT SARS-CoV-2, NICPR13, NICPR20, NICPR21, NICPR293 & NICPR294** | **HLA-A11:01** |
| **28** | **66** | **AIHVSGTTK** | **WT SARS-CoV-2, NICPR13, NICPR20, NICPR21, NICPR293 & NICPR294** | **HLA-A11:01** |
| **29** | **741** | **LQYGSTQLK** | **NICPR13, NICPR20, NICPR21, NICPR293 & NICPR294** | **HLA-A11:01** |
| **30** | **20** | **RTYTNSFTR** | **NICPR20, NICPR21, NICPR293 & NICPR294** | **HLA-A11:01** |
| **31** | **264** | **YYYLQPRTF** | **WT SARS-CoV-2, NICPR13, NICPR20, NICPR21, NICPR293 & NICPR294** | **HLA-A24:02** |
| **32** | **327** | **RFPNITNLF** | **WT SARS-CoV-2, NICPR13, NICPR20, NICPR21, NICPR293 & NICPR294** | **HLA-A24:02** |
| **33** | **53** | **LFLPSNVTW** | **WT SARS-CoV-2, NICPR13, NICPR20, NICPR21, NICPR293 & NICPR294** | **HLA-A24:02** |
| **34** | **1210** | **QYIKWPWYL** | **WT SARS-CoV-2, NICPR13, NICPR20, NICPR21, NICPR293 & NICPR294** | **HLA-A24:02** |
| **35** | **36** | **YYPDKVFSV** | **WT SARS-CoV-2, NICPR13, NICPR20, NICPR21, NICPR293 & NICPR294** | **HLA-A24:02** |
| **36** | **168** | **EYVSQPFLL** | **WT SARS-CoV-2, NICPR20, NICPR21, NICPR293 & NICPR294** | **HLA-A24:02** |
| **37** | **265** | **YYLQPRTFL** | **WT SARS-CoV-2, NICPR13, NICPR20, NICPR21, NICPR293 & NICPR294** | **HLA-A24:02** |
| **38** | **1067** | **TYVPAQEKF** | **WT SARS-CoV-2, NICPR13, NICPR20, NICPR21, NICPR293 & NICPR294** | **HLA-A24:02** |
| **39** | **21** | **TQLPPAYTF** | **WT SARS-CoV-2 and NICPR13** | **HLA-A24:02** |
| **40** | **89** | **VYFASTEKI** | **WT SARS-CoV-2, NICPR13, NICPR20, NICPR21, NICPR293 & NICPR294** | **HLA-A24:02** |
| **41** | **166** | **TYVSQPFLM** | **WT SARS-CoV-2 & NICPR13** | **HLA-A24:02** |
| **42** | **1208** | **YYIKWPWYI** | **WT SARS-CoV-2, NICPR13, NICPR20, NICPR21, NICPR293 & NICPR294** | **HLA-A24:02** |
| **43** | **157** | **VYSSANNTF** | **WT SARS-CoV-2, NICPR20, NICPR21, NICPR293 & NICPR294** | **HLA-A24:02** |
| **44** | **363** | **DYLYNSASF** | **WT SARS-CoV-2** | **HLA-A24:02** |
| **45** | **874** | **QYTSALLTI** | **WT SARS-CoV-2, NICPR13, NICPR20, NICPR21, NICPR293 & NICPR294** | **HLA-A24:02** |
| **46** | **635** | **WYSTGSNVF** | **WT SARS-CoV-2, NICPR13, NICPR20, NICPR21, NICPR293 & NICPR294** | **HLA-A24:02** |
| **47** | **461** | **KPRDISTEI** | **WT SARS-CoV-2, NICPR13, NICPR20, NICPR21, NICPR293 & NICPR294** | **HLA-B07:02** |
| **48** | **37** | **YPDKRSSVL** | **WT SARS-CoV-2, NICPR13, NICPR20, NICPR21, NICPR293 & NICPR294** | **HLA-B07:02, HLA-B08:01** |
| **49** | **1054** | **FPAPHGVVF** | **WT SARS-CoV-2, NICPR13, NICPR20, NICPR21, NICPR293 & NICPR294** | **HLA-B07:02, Repeat 3 B35:01, B51:01** |
| **50** | **813** | **KPSKRSFIL** | **WT SARS-CoV-2, NICPR13, NICPR20, NICPR21, NICPR293 & NICPR294** | **HLA-B07:02** |
| **51** | **318** | **RPTESIVRF** | **WT SARS-CoV-2, NICPR13, NICPR20, NICPR21, NICPR293 & NICPR294** | **HLA-B07:02** |
| **52** | **680** | **TPRRARSVA** | **WT SARS-CoV-2** | **HLA-B07:02** |
| **53** | **1016** | **RAAEIRASL** | **WT SARS-CoV-2, NICPR13, NICPR20, NICPR21, NICPR293 & NICPR294** | **HLA-B07:02** |
| **54** | **577** | **AVRDPQTIL** | **WT SARS-CoV-2, NICPR13, NICPR20, NICPR21, NICPR293 & NICPR294** | **HLA-B07:02** |
| **55** | **215** | **LPQGFSAPL** | **WT SARS-CoV-2, NICPR13, NICPR20, NICPR21, NICPR293 & NICPR294** | **HLA-B07:02** |
| **56** |  | **KPINLGRDL (200)** | **Only in NICPR294** | **HLA-B07:02** |
| **57** | **450** | **YLYRRKSNL/ YLYRRKSKL** | **WT SARS-CoV-2 & NICPR13/ NICPR293, NICPR21, NICPR20** | **HLA-B08:01** |
| **58** | **1054** | **FPQPHGVVF** | **WT SARS-CoV-2** | **HLA-B08:01, HLA-C04:01** |
| **59** | **407** | **RQIAPGQTI/**  **SQIAPGQTI** | **WT SARS-CoV-2, NICPR13/ NICPR20, NICPR21, NICPR293, NICPR294** | **HLA-B13:02, HLA-B52:01** |
| **60** | **132** | **FQFDPFLGV** | **WT SARS-CoV-2 only** | **HLA-B13:02** |
| **61** | **627** | **HQLTPTWRV** | **WT SARS-CoV-2, NICPR13, NICPR20, NICPR21, NICPR293 & NICPR294** | **HLA-B13:02** |
| **62** | **1181** | **IQKEILNEV** | **WT SARS-CoV-2, NICPR13, NICPR20, NICPR21, NICPR293 & NICPR294** | **HLA-B13:02** |
| **63** | **985** | **RLVEAEVQI** | **WT SARS-CoV-2, NICPR13, NICPR20, NICPR21, NICPR293 & NICPR294** | **HLA-B13:02** |
| **64** | **993** | **VQIDRLITL** | **WT SARS-CoV-2, NICPR13, NICPR20, NICPR21, NICPR293 & NICPR294** | **HLA-B13:02** |
| **65** | **780** | **TQFAQVKQI** | **WT SARS-CoV-2, NICPR13, NICPR20, NICPR21, NICPR293 & NICPR294** | **HLA-B13:02** |
| **66** | **1201** | **DELGKYEQY** | **WT SARS-CoV-2, NICPR13, NICPR293, NICPR294** | **HLA-B18:01** |
| **67s** | **919** | **YENQIANQF** | **WT SARS-CoV-2, NICPR13, NICPR20, NICPR21, NICPR293 & NICPR294** | **HLA-B18:01** |
| **68** | **781** | **QEVQVKQIY** | **WT SARS-CoV-2, NICPR13, NICPR20, NICPR21, NICPR293 & NICPR294** | **HLA-B18:01** |
| **69** | **869** | **DEMQYTSAL** | **WT SARS-CoV-2, NICPR13, NICPR20, NICPR21, NICPR293 & NICPR294** | **HLA-B18:01** |
| **70** | **774** | **VEQDTQEVF** | **WT SARS-CoV-2, NICPR13, NICPR20, NICPR21, NICPR293 & NICPR294** | **HLA-B18:01** |
| **71** | **307** | **VEKGIYQTF** | **WT SARS-CoV-2, NICPR13, NICPR20, NICPR21, NICPR293 & NICPR294** | **HLA-B18:01** |
| **72** | **666** | **IPIGAGISY** | **WT SARS-CoV-2, NICPR13, NICPR20, NICPR21, NICPR293 & NICPR294** | **HLA-B35:01** |
| **76** | **80** | **NPVLPFNVY** | **WT SARS-CoV-2, NICPR13, NICPR20, NICPR21, NICPR293 & NICPR294** | **HLA-B35:01** |
| **77** | **896** | **LPFAMQMAY** | **WT SARS-CoV-2, NICPR13, NICPR20, NICPR21, NICPR293 & NICPR294** | **HLA-B35:01** |
| **78** | **699** | **MSAENSVAY** | **WT SARS-CoV-2, NICPR13, NICPR20, NICPR21, NICPR293 & NICPR294** | **HLA-B35:01, HLA-B46:01** |
| **79** | **1113** | **EPITTDNTF** | **WT SARS-CoV-2, NICPR13, NICPR20, NICPR21, NICPR293 & NICPR294** | **HLA-B35:01** |
| **80** | **1264** | **EPVGVKLHY** | **WT SARS-CoV-2, NICPR13, NICPR20, NICPR21, NICPR293 & NICPR294** | **HLA-B35:01** |
| **81** | **500** | **QPTNGVQPY** | **WT SARS-CoV-2** | **HLA-B35:01** |
| **82** | **37** | **YPDKVSSVL** | **WT SARS-CoV-2, NICPR13, NICPR20, NICPR21, NICPR293 & NICPR294** | **HLA-B35:01, HLA-C04:01** |
| **83** | **487** | **CPYFPLQSY** | **WT SARS-CoV-2** | **HLA-B35:01** |
| **84** | **898** | **IPFAMQMAF** | **WT SARS-CoV-2, NICPR13, NICPR20, NICPR21, NICPR293 & NICPR294** | **HLA-B35:01** |
| **85** |  | **YPGDSSSSW** | **NICPR20, NICPR21, NICPR293** | **HLA-B35:01** |
| **86** | **1018** | **AEISANLAA** | **WT SARS-CoV-2, NICPR13, NICPR20, NICPR21, NICPR293 & NICPR294** | **HLA-B40:06** |
| **87** | **152** | **MESEFRVSA/**  **MESELRVSA** | **WT SARS-CoV-2, NICPR13, NICPR293, NICPR294/ NICPR20, NICPR21** | **HLA-B40:06** |
| **88** | **1054** | **FSAPHGVVF** | **WT SARS-CoV-2, NICPR13, NICPR20, NICPR21, NICPR293 & NICPR294** | **HLA-B46:01** |
| **89** | **1130** | **VVIGNNTVY** | **WT SARS-CoV-2, NICPR13, NICPR20, NICPR21, NICPR293 & NICPR294** | **HLA-B46:01** |
| **90** | **27** | **YSFTRGVYY** | **WT SARS-CoV-2, NICPR13, NICPR20, NICPR21, NICPR293 & NICPR294** | **HLA-B46:01** |
| **91** | **497** | **YGFQPTVGY** | **WT SARS-CoV-2** | **HLA-B46:01** |
| **92** | **687** | **RASQSIIAY** | **WT SARS-CoV-2, NICPR13, NICPR20, NICPR21, NICPR293 & NICPR294** | **HLA-B46:01** |
| **93** | **590** | **TPFGGVSVI** | **WT SARS-CoV-2, NICPR13, NICPR20, NICPR21, NICPR293 & NICPR294** | **HLA-B51:01** |
| **94** | **576** | **DAVPQTLEI** | **WT SARS-CoV-2, NICPR13, NICPR20, NICPR21, NICPR293 & NICPR294** | **HLA-B51:01** |
| **95** | **461** | **KPFDISTEI** | **WT SARS-CoV-2, NICPR13, NICPR20, NICPR21, NICPR293 & NICPR294** | **HLA-B51:01** |
| **96** | **714** | **IPTNFTISV** | **WT SARS-CoV-2, NICPR13, NICPR20, NICPR21, NICPR293 & NICPR294** | **HLA-B51:01** |
| **97** | **21** | **TQLPPTNSF** | **WT SARS-CoV-2, NICPR13** | **HLA-B52:01** |
| **98** | **212** | **VLPQGFSAL/**  **GLPQGFSAL** | **WT SARS-CoV-2/ NICPR20, NICPR21, NICPR293 & NICPR294** | **HLA-C01:02** |
| **99** | **525** | **TVPKKSTNL** | **WT SARS-CoV-2, NICPR13, NICPR20, NICPR21, NICPR293 & NICPR294** | **HLA-C01:02** |
| **100** | **379** | **YSPTKLNDL** | **WT SARS-CoV-2, NICPR13, NICPR20, NICPR21, NICPR293 & NICPR294** | **HLA-C01:02** |
| **101** | **1257** | **KFDDSEPVL** | **WT SARS-CoV-2, NICPR13, NICPR20, NICPR21, NICPR293 & NICPR294** | **HLA-C04:01** |
| **102** | **1137** | **NYDPLQPEL** | **WT SARS-CoV-2, NICPR13, NICPR20, NICPR21, NICPR293 & NICPR294** | **HLA-C04:01** |
| **103** | **75** | **TFDNPVLPF** | **WT SARS-CoV-2, NICPR13, NICPR20, NICPR21, NICPR293 & NICPR294** | **HLA-C04:01** |
| **104** | **1139** | **VYDPLQPEL** | **WT SARS-CoV-2, NICPR13, NICPR20, NICPR21, NICPR293 & NICPR294** | **HLA-C04:01** |
| **105** | **324** | **SRFPNITNL** | **WT SARS-CoV-2, NICPR13, NICPR20, NICPR21, NICPR293 & NICPR294** | **HLA-C06:02, HLA-C07:01** |

**Table TS9: B cell epitope prediction**

| **S.No.** | **Position** | **Epitope Sequence** | **Samples** | **Score** |
| --- | --- | --- | --- | --- |
| **4** | **879** | **AGTITSGWTFGAGAAL** | **WT SARS-CoV-2, NICPR13, NICPR20, NICPR21, NICPR293 & NICPR294** | **0.97** |
| **6** | **594** | **GVSVITPGTNTSNQVA** | **WT SARS-CoV-2, NICPR13, NICPR20, NICPR21, NICPR293 & NICPR294** | **0.95** |
| **8** | **257** | **GWTAGAAAYYVGYLQP / SWTAGAAAYYVGYLQP (254)** | **Present in WT SARS-CoV-2, NICPR294, NICPR21/ WT SARS-CoV-2, NICPR13, NICPR20, NICPR21, NICPR293 & NICPR294** | **0.95** |
| **10** | **1112** | **PQIITTDNTFVSGNCD** | **WT SARS-CoV-2, NICPR13, NICPR20, NICPR21, NICPR293 & NICPR294** | **0.95** |
| **12** | **245** | **HRSYLTPGDSSSGWTA / HRSYLTPGDSSSSWTA** | **WT SARS-CoV-2, NICPR294, NICPR21/ WT SARS-CoV-2, NICPR13, NICPR20, NICPR21, NICPR293 & NICPR294** | **0.92** |
| **14** | **1180** | **QKEIDRLNEVAKNLNE** | **WT SARS-CoV-2, NICPR13, NICPR20, NICPR21, NICPR293 & NICPR294** | **0.92** |
| **16** | **476/470** | **GSTPCNGVEGFNCYFP/ TEIYQAGSTPCNGVEG** | **Only in WT SARS-CoV-2** | **0.91** |
| **18** | **307** | **TVEKGIYQTSNFRVQP** | **WT SARS-CoV-2, NICPR13, NICPR20, NICPR21, NICPR293 & NICPR294** | **0.91** |
| **20** | **648** | **GCLIGAEHVNNSYECD** | **WT SARS-CoV-2** | **0.9** |
| **22** | **492** | **LQSYGFQPTNGVGYQP** | **WT SARS-CoV-2 only** | **0.9** |
| **24** | **1247** | **CCSCGSCCKFDEDDSE** | **WT SARS-CoV-2, NICPR13, NICPR20, NICPR21, NICPR293 & NICPR294** | **0.9** |
| **26** | **470** | **TEIYQAGSTPCNGVEG** | **Only in WT SARS-CoV-2** | **0.89** |
| **28** | **236** | **TRFQTLLALHRSYLTP** | **WT SARS-CoV-2, NICPR13, NICPR20, NICPR21, NICPR293 & NICPR294** | **0.89** |
| **30** | **1206** | **YEQYIKWPWYIWLGFI /**  **YEQYIKWPWYIWLVFI (1201)** | **WT SARS-CoV-2, NICPR13, NICPR20, NICPR21, & NICPR294 / WT SARS-CoV-2, NICPR13, NICPR20, NICPR21, NICPR293 & NICPR294** | **0.89** |
| **32** | **1058** | **HGVVFLHVTYVPAQEK** | **WT SARS-CoV-2, NICPR13, NICPR20, NICPR21, NICPR293 & NICPR294** | **0.89** |
| **34** | **931** | **IGKIQDSLSSTASALG** | **WT SARS-CoV-2, NICPR13, NICPR20, NICPR21, NICPR293 & NICPR294** | **0.88** |
| **36** | **898** | **FAMQMAYRFNGIGVTQ** | **WT SARS-CoV-2, NICPR13, NICPR20, NICPR21, NICPR293 & NICPR294** | **0.88** |
| **38** | **1064** | **HVTYVPAQEKNFTTAP** | **WT SARS-CoV-2, NICPR13, NICPR20, NICPR21, NICPR293 & NICPR294** | **0.88** |
| **40** | **786** | **KQIYKTPPIKDFGGFN** | **Only in WT SARS-CoV-2** | **0.87** |
| **42** | **525** | **CGPKKSTNLVKNKCVN** | **WT SARS-CoV-2, NICPR13, NICPR20, NICPR21, NICPR293 & NICPR294** | **0.86** |
| **44** | **464** | **FERDISTEIYQAGSTP / FERDISTEIYQAGNKP** | **WT SARS-CoV-2 only** | **0.86** |
| **46** | **266** | **YVGYLQPRTFLLKYNE** | **WT SARS-CoV-2, NICPR13, NICPR20, NICPR21, NICPR293 & NICPR294** | **0.86** |
| **48** | **151** | **SWMESEFRVYSSANNC** | **WT SARS-CoV-2, NICPR294, NICPR21** | **0.86** |
| **50** | **1240** | **CCSCLKGCCSCGSCCK** | **WT SARS-CoV-2, NICPR13, NICPR20, NICPR21, NICPR293 & NICPR294** | **0.86** |
| **52** | **732** | **TKTSVDCTMYICGDST** | **WT SARS-CoV-2, NICPR13, NICPR20, NICPR21, NICPR293 & NICPR294** | **0.85** |
| **54** | **406** | **EVRQIAPGQTGKIADY / EVRQIAPGQTGNIADY (394)** | **WT SARS-CoV-2/ NICPR21** | **0.85** |
| **56** | **391** | **CFTNVYADSFVIRGDE / CFTNVYADSFVIRGNE** | **WT SARS-CoV-2, NICPR21 and / WT SARS-CoV-2, NICPR13, NICPR20, NICPR21, NICPR293 & NICPR294** | **0.85** |
| **58** | **1051** | **MSFPQSAPHGVVFLHV** | **WT SARS-CoV-2, NICPR13, NICPR20, NICPR21, NICPR293 & NICPR294** | **0.85** |
| **60** | **97** | **KSNIIRGWIFGTTLDS** | **WT SARS-CoV-2, NICPR13, NICPR20, NICPR21, NICPR293 & NICPR294** | **0.84** |
| **62** | **739** | **TMYICGDSTECSNLLL** | **WT SARS-CoV-2, NICPR13, NICPR20, NICPR21, NICPR293 & NICPR294** | **0.84** |
| **64** | **630** | **TPTWRVYSTGSNVFQT** | **WT SARS-CoV-2, NICPR13, NICPR20, NICPR21, NICPR293 & NICPR294** | **0.84** |
| **66** | **415** | **TGKIADYNYKLPDDFT** | **WT SARS-CoV-2 only** | **0.84** |
| **68** | **200** | **YFKIYSKHTPINLVRD/**  **YFKIYSKHTPINLGRD/**  **YFKIYSKHTPVNLGRD/**  **YFKIYSKHTPIIVREP/**  **FKNIDGYFKIYSKHTP** | **WT SARS-CoV-2 only/ NICPR294 (195)/ NICPR20 (197), NICPR293 (195)/ NICPR13 (186)/ WT SARS-CoV-2, NICPR13, NICPR20, NICPR21, NICPR293 & NICPR294** | **0.84** |
| **70** | **1234** | **LCCMTSCCSCLKGCCS** | **WT SARS-CoV-2, NICPR13, NICPR20, NICPR21, NICPR293 & NICPR294** | **0.84** |
| **72** | **1084** | **DGKAHFPREGVFVSNG** | **WT SARS-CoV-2, NICPR13, NICPR20, NICPR21, NICPR293 & NICPR294** | **0.84** |
| **74** | **847** | **RDLICAQKFNGLTVLP/**  **RDLICAQKFKGLTVLP (835)** | **WT SARS-CoV-2, NICPR20, NICPR21, NICPR293 & NICPR294/ NICPR13 (835)** | **0.83** |
| **76** | **604** | **TSNQVAVLYQDVNCTE** | **WT SARS-CoV-2 only** | **0.83** |
| **78** | **280** | **NENGTITDAVDCALDP** | **WT SARS-CoV-2, NICPR13, NICPR20, NICPR21, NICPR293 & NICPR294** | **0.83** |
| **80** | **1195** | **ESLIDLQELGKYEQYI** | **WT SARS-CoV-2, NICPR13, NICPR293 & NICPR294** | **0.83** |
| **82** | **1129** | **VIGIVNNTVYDPLQPE** | **WT SARS-CoV-2, NICPR13, NICPR20, NICPR21, NICPR293 & NICPR294** | **0.83** |
| **84** | **70** | **VSGTNGTKRFDNPVLP/**  **ISGTNGTKRFDNPVLP/**  **HXXSGTNGTKRFDNPV** | **WT SARS-CoV-2, NICPR20 & NICPR21/ NICPR13 (68) & NICPR293 (65)/ NICPR294 (63)** | **0.82** |
| **86** | **689** | **SQSIIAYTMSLGAENS** | **WT SARS-CoV-2, NICPR13, NICPR20, NICPR21, NICPR293 & NICPR294** | **0.82** |
| **88** | **564** | **QFGRDIADTTDAVRDP** | **WT SARS-CoV-2, NICPR13, NICPR20, NICPR21, NICPR293 & NICPR294** | **0.82** |
| **90** | **329** | **FPNITNLCPFGEVFNA/**  **FPNITNLCPFDEVFNA/**  **FPNITNLCPFHEVFNA** | **WT SARS-CoV-2/ NICPR294 (324), NICPR13 (317)/ NICPR21 (326), NICPR20 (326)** | **0.82** |
| **92** | **347** | **FASVYAWNRKRISNCV** | **WT SARS-CoV-2, NICPR13, NICPR20, NICPR21, NICPR293 & NICPR294** | **0.81** |
| **94** | **1041** | **DFCGKGYHLMSFPQSA** | **WT SARS-CoV-2, NICPR13, NICPR20, NICPR21, NICPR293 & NICPR294** | **0.81** |
| **96** | **803** | **SQILPDPSKPSKRSFI** | **WT SARS-CoV-2, NICPR13, NICPR20, NICPR21, NICPR293 & NICPR294** | **0.8** |
| **98** | **674** | **YQTQTNSPRRARSVAS/**  **IGAGICASYQTQTNSP (666)** | **WT SARS-CoV-2 only** | **0.8** |
| **100** | **583** | **EILDITPCSFGGVSVI** | **WT SARS-CoV-2, NICPR13, NICPR20, NICPR21, NICPR293 & NICPR294** | **0.8** |
| **102** | **49** | **HSTQDLFLPFFSNVTW** | **WT SARS-CoV-2, NICPR13, NICPR20, NICPR21, NICPR293 & NICPR294** | **0.8** |
| **104** | **320** | **VQPTESIVRFPNITNL** | **WT SARS-CoV-2, NICPR13, NICPR20, NICPR21, NICPR293 & NICPR294** | **0.8** |
| **106** | **288** | **AVDCALDPLSETKCTL** | **WT SARS-CoV-2, NICPR13, NICPR20, NICPR21, NICPR293 & NICPR294** | **0.8** |
| **108** | **194** | **FKNIDGYFKIYSKHTP** | **WT SARS-CoV-2, NICPR13, NICPR20, NICPR21, NICPR293 & NICPR294** | **0.8** |
| **110** | **124** | **TNVVIKVCEFQFCNDP** | **WT SARS-CoV-2, NICPR13, NICPR20, NICPR21, NICPR293 & NICPR294** | **0.8** |
| **112** | **829** | **ADAGFIKQYGDCLGDI** | **WT SARS-CoV-2, NICPR13, NICPR20, NICPR21, NICPR293 & NICPR294** | **0.79** |
| **114** | **360** | **NCVADYSVLYNSASFS/**  **NCVADYSVLYNFAPFF/**  **RKRISNCVADYSVLYN** | **WT SARS-CoV-2/ NICPR294 (355), NICPR21 (357), NICPR293 (355), NICPR20 (357)/ NICPR13 (343)** | **0.79** |
| **116** | **19** | **TTRTQLPPAYTNSFTR/**  **VSSQCVNLTTRTQLPP (11)** | **WT SARS-CoV-2 and NICPR13 only/ WT SARS-CoV-2 (11) and NICPR13 (11) only** | **0.79** |
| **118** | **159** | **VYSSANNCTFEYVSQP** | **WT SARS-CoV-2, NICPR20, NICPR21, NICPR293 & NICPR294** | **0.78** |
| **120** | **107** | **GTTLDSKTQSLLIVNN** | **WT SARS-CoV-2, NICPR13, NICPR20, NICPR21, NICPR293 & NICPR294** | **0.78** |
| **122** | **994** | **DRLITGRLQSLQTYVT** | **WT SARS-CoV-2, NICPR13, NICPR20, NICPR21, NICPR293 & NICPR294** | **0.77** |
| **124** | **719** | **TISVTTEILPVSMTKT** | **WT SARS-CoV-2, NICPR13, NICPR20, NICPR21, NICPR293 & NICPR294** | **0.77** |
| **126** | **657** | **NNSYECDIPIGAGICA** | **WT SARS-CoV-2, NICPR13, NICPR20, NICPR21, NICPR293 & NICPR294** | **0.77** |
| **128** | **501** | **NGVGYQPYRVVVLSFE/**  **YGVGYQPYRVVVLSFE (498)** | **WT SARS-CoV-2 only/ NICPR20 (498)** | **0.77** |
| **130** | **1227** | **IVMVTIMLCCMTSCCS** | **WT SARS-CoV-2, NICPR13, NICPR20, NICPR21, NICPR293 & NICPR294** | **0.77** |
| **132** | **941** | **TASALGKLQDVVNQNA/**  **TASALGKLQDVVNHNA** | **WT SARS-CoV-2/ NICR294, NICPR13, NICPR21, NICPR293, NICPR20.** | **0.76** |
| **134** | **838** | **GDCLGDIAARDLICAQ** | **WT SARS-CoV-2, NICPR13, NICPR20, NICPR21, & NICPR294** | **0.76** |
| **136** | **709** | **NNSIAIPTNFTISVTT** | **WT SARS-CoV-2, NICPR13, NICPR20, NICPR21, NICPR293 & NICPR294** | **0.76** |
| **138** | **695** | **YTMSLGAENSVAYSNN** | **WT SARS-CoV-2, NICPR13, NICPR20, NICPR21, NICPR293 & NICPR294** | **0.76** |
| **140** | **374** | **FSTFKCYGVSPTKLND** | **WT SARS-CoV-2 only** | **0.76** |
| **142** | **301** | **CTLKSFTVEKGIYQTS** | **WT SARS-CoV-2, NICPR13, NICPR20, NICPR21, NICPR293 & NICPR294** | **0.76** |
| **144** | **251** | **PGDSSSGWTAGAAAYY** | **WT SARS-CoV-2, NICPR294 & NICPR13 only.** | **0.76** |
| **146** | **1146** | **DSFKEELDKYFKNHTS** | **WT SARS-CoV-2, NICPR13, NICPR20, NICPR21, NICPR293 & NICPR294** | **0.76** |
| **148** | **778** | **TQEVFAQVKQIYKTPP** | **WT SARS-CoV-2, NICPR13, NICPR20, NICPR21, NICPR293 & NICPR294** | **0.75** |
| **150** | **666** | **IGAGICASYQTQTNSP** | **WT SARS-CoV-2 only** | **0.75** |
| **152** | **1139** | **DPLQPELDSFKEELDK** | **WT SARS-CoV-2, NICPR13, NICPR20, NICPR21, NICPR293 & NICPR294** | **0.75** |
| **154** | **815** | **RSFIEDLLFNKVTLAD** | **WT SARS-CoV-2, NICPR13, NICPR20, NICPR21, NICPR293 & NICPR294** | **0.74** |
| **156** | **765** | **RALTGIAVEQDKNTQE** | **WT SARS-CoV-2, NICPR13, NICPR20, NICPR21, NICPR293 & NICPR294** | **0.74** |
| **158** | **366** | **SVLYNSASFSTFKCYG** | **WT SARS-CoV-2 only** | **0.74** |
| **160** | **34** | **RGVYYPDKVFRSSVLH** | **WT SARS-CoV-2, NICPR13, NICPR20, NICPR21, NICPR293 & NICPR294** | **0.74** |
| **162** | **1103** | **FVTQRNFYEPQIITTD** | **WT SARS-CoV-2, NICPR13, NICPR20, NICPR21, NICPR293 & NICPR294** | **0.74** |
| **164** | **988** | **EAEVQIDRLITGRLQS** | **WT SARS-CoV-2, NICPR13, NICPR20, NICPR21, NICPR293 & NICPR294** | **0.73** |
| **166** | **860** | **VLPPLLTDEMIAQYTS** | **WT SARS-CoV-2, NICPR13, NICPR20, NICPR21, NICPR293 & NICPR294** | **0.73** |
| **168** | **547** | **TGTGVLTESNKKFLPF/**  **NGLKGTGVLTESNKKF (s13/532)** | **WT SARS-CoV-2, NICPR13, NICPR20, NICPR21, NICPR293 & NICPR294/ NICPR13 (532)** | **0.73** |
| **170** | **484** | **EGFNCYFPLQSYGFQP/**  **AGFNCYFPLQSYGFRP (479)** | **WT SARS-CoV-2 and / NICPR293 (479)** | **0.73** |
| **172** | **136** | **CNDPFLGVYYHKNNKS** | **WT SARS-CoV-2 only** | **0.73** |
| **174** | **1166** | **LGDISGINASVVNIQK** | **WT SARS-CoV-2, NICPR13, NICPR20, NICPR21, NICPR293 & NICPR294** | **0.73** |
| **176** | **1009** | **TQQLIRAAEIRASANL** | **WT SARS-CoV-2, NICPR13, NICPR20, NICPR21, NICPR293 & NICPR294** | **0.73** |
| **178** | **88** | **DGVYFASTEKSNIIRG** | **WT SARS-CoV-2, NICPR13, NICPR20, NICPR21, NICPR293 & NICPR294** | **0.72** |
| **180** | **747** | **TECSNLLLQYGSFCTQ** | **WT SARS-CoV-2, NICPR13, NICPR20, NICPR21, NICPR293 & NICPR294** | **0.72** |
| **182** | **1094** | **VFVSNGTHWFVTQRNF** | **WT SARS-CoV-2, NICPR13, NICPR20, NICPR21, NICPR293 & NICPR294** | **0.72** |
| **184** | **953** | **NQNAQALNTLVKQLSS/**  **NHNAQALNTLVKQLSS** | **WT SARS-CoV-2/ NICPR294, NICPR13, NICPR21, NICPR293 & NICPR20** | **0.71** |
| **186** | **636** | **YSTGSNVFQTRAGCLI** | **WT SARS-CoV-2, NICPR13, NICPR20, NICPR21, NICPR293 & NICPR294** | **0.71** |
| **188** | **445** | **VGGNYNYLYRLFRKSN/**  **KVSGNYNYLYRLFRKS (432)** | **WT SARS-CoV-2/ NICPR13(432), NICPR21(441), NICPR293(439) & NICPR20(441).** | **0.71** |
| **190** | **430** | **TGCVIAWNSNNLDSKV/**  **TGCVIAWNSNKLDSKV** | **WT SARS-CoV-2/ NICPR13 (418), NICPR21 (427), NICPR20 (427), NICPR293 (425), NICPR294(425).** | **0.71** |
| **192** | **423** | **YKLPDDFTGCVIAWNS** | **WT SARS-CoV-2 only** | **0.71** |
| **194** | **1001** | **LQSLQTYVTQQLIRAA** | **WT SARS-CoV-2, NICPR13, NICPR20, NICPR21, NICPR293 & NICPR294** | **0.71** |
| **196** | **612** | **YQDVNCTEVPVAIHAD** | **WT SARS-CoV-2 only** | **0.7** |
| **198** | **25** | **PPAYTNSFTRGVYYPD/**  **TRTQSYTNSFTRGVYY** | **WT SARS-CoV-2 & NICPR13/ NICPR294 (20), NICPR21 (20), NICPR20 (20), NICPR293 (20)** | **0.7** |
| **200** | **1201** | **PWYIWLGFIAGLIAIV** | **WT SARS-CoV-2, NICPR13, NICPR20, NICPR21, & NICPR294** | **0.7** |
| **202** | **1121** | **FVSGNCDVVIGIVNNT** | **WT SARS-CoV-2, NICPR13, NICPR20, NICPR21, NICPR293 & NICPR294** | **0.7** |
| **204** | **890** | **AGAALQIPFAMQMAYR** | **WT SARS-CoV-2, NICPR13, NICPR20, NICPR21, NICPR293 & NICPR294** | **0.69** |
| **206** | **400** | **FVIRGDEVRQIAPGQT** | **WT SARS-CoV-2 Only** | **0.69** |
| **208** | **1153** | **DKYFKNHTSPDVDLGD** | **WT SARS-CoV-2, NICPR13, NICPR20, NICPR21, NICPR293 & NICPR294** | **0.69** |
| **210** | **1078** | **APAICHDGKAHFPREG** | **WT SARS-CoV-2, NICPR13, NICPR20, NICPR21, NICPR293 & NICPR294** | **0.69** |
| **212** | **294** | **DPLSETKCTLKSFTVE** | **WT SARS-CoV-2, NICPR13, NICPR20, NICPR21, NICPR293 & NICPR294** | **0.68** |
| **214** | **573** | **TDAVRDPQTLEILDIT** | **WT SARS-CoV-2, NICPR13, NICPR20, NICPR21, NICPR293 & NICPR294** | **0.67** |
| **216** | **910** | **GVTQNVLYENQKLIAN** | **WT SARS-CoV-2, NICPR13, NICPR20, NICPR21, NICPR293 & NICPR294** | **0.66** |
| **218** | **771** | **AVEQDKNTQEVFAQVK** | **WT SARS-CoV-2, NICPR13, NICPR20, NICPR21, NICPR293 & NICPR294** | **0.66** |
| **220** | **511** | **VVLSFELLHAPATVCG** | **WT SARS-CoV-2, NICPR13, NICPR20, NICPR21, NICPR293 & NICPR294** | **0.66** |
| **222** | **3** | **VFLVLLPLVSSQCVNL** | **WT SARS-CoV-2, NICPR13, NICPR20, NICPR21, NICPR293 & NICPR294** | **0.66** |
| **224** | **1159** | **HTSPDVDLGDISGINA** | **WT SARS-CoV-2, NICPR13, NICPR20, NICPR21, NICPR293 & NICPR294** | **0.66** |
| **226** | **868** | **EMIAQYTSALLAGTIT** | **WT SARS-CoV-2, NICPR13, NICPR20, NICPR21, NICPR293 & NICPR294** | **0.65** |
| **228** | **457** | **RKSNLKPFERDISTEI** | **WT SARS-CoV-2, NICPR294 & NICPR13 only** | **0.65** |
| **230** | **224** | **EPLVDLPIGINITRFQ** | **WT SARS-CoV-2, NICPR13, NICPR20, NICPR21, NICPR293 & NICPR294** | **0.65** |
| **232** | **174** | **PFLMDLEGKQGNFKNL/**  **NCLMDLEGKQGNFKNL (160)** | **WT SARS-CoV-2, NICPR20, NICPR21, NICPR293 & NICPR294/ NICPR13 (160)** | **0.65** |
| **234** | **11** | **VSSQCVNLTTRTQLPP** | **WT SARS-CoV-2 and NICPR13 only** | **0.65** |
| **236** | **975** | **SVLNDILSRLDKVEAE** | **WT SARS-CoV-2, NICPR20, NICPR21, NICPR293 & NICPR294** | **0.64** |
| **238** | **1257** | **DEDDSEPVLKGVKLHY** | **WT SARS-CoV-2, NICPR13, NICPR20, NICPR21, NICPR293 & NICPR294** | **0.64** |
| **240** | **964** | **KQLSSNFGAISSVLND** | **WT SARS-CoV-2 only** | **0.63** |
| **242** | **82** | **PVLPFNDGVYFASTEK** | **WT SARS-CoV-2, NICPR13, NICPR20, NICPR21, NICPR293 & NICPR294** | **0.63** |
| **244** | **76** | **TKRFDNPVLPFNDGVY** | **WT SARS-CoV-2, NICPR13, NICPR20, NICPR21, NICPR293 & NICPR294** | **0.63** |
| **246** | **384** | **PTKLNDLCFTNVYADS** | **WT SARS-CoV-2, NICPR13, NICPR20, NICPR21, NICPR293 & NICPR294** | **0.62** |
| **248** | **43** | **FRSSVLHSTQDLFLPF** | **WT SARS-CoV-2, NICPR13, NICPR20, NICPR21, NICPR293 & NICPR294** | **0.61** |
| **250** | **1032** | **CVLGQSKRVDFCGKGY** | **WT SARS-CoV-2, NICPR13, NICPR20, NICPR21, NICPR293 & NICPR294** | **0.61** |
| **252** | **1026** | **ATKMSECVLGQSKRVD** | **WT SARS-CoV-2, NICPR13, NICPR20, NICPR21, NICPR293 & NICPR294** | **0.61** |
| **254** | **1020** | **ASANLAATKMSECVLG** | **WT SARS-CoV-2, NICPR13, NICPR20, NICPR21, NICPR293 & NICPR294** | **0.6** |
| **256** | **210** | **INLVRDLPQGFSALEP** | **WT SARS-CoV-2 only** | **0.59** |
| **258** | **757** | **GSFCTQLNRALTGIAV** | **WT SARS-CoV-2 only** | **0.57** |
| **260** | **182** | **KQGNFKNLREFVFKNI** | **WT SARS-CoV-2, NICPR13, NICPR20, NICPR21, NICPR293 & NICPR294** | **0.57** |
| **262** | **118** | **LIVNNATNVVIKVCEF** | **WT SARS-CoV-2, NICPR13, NICPR20, NICPR21, NICPR293 & NICPR294** | **0.55** |
| **264** | **922** | **LIANQFNSAIGKIQDS** | **WT SARS-CoV-2, NICPR13, NICPR20, NICPR21, NICPR293 & NICPR294** | **0.54** |
| **266** | **792** | **PPIKDFGGFNFSQILP** | **WT SARS-CoV-2 only** | **0.52** |

**Table TS10: B cell epitope prediction**

| **S.No.** | **Position** | **Epitope Sequence** | **Samples** | **Score** |
| --- | --- | --- | --- | --- |
| **4** | **879** | **AGTITSGWTFGAGAAL** | **WT SARS-CoV-2, NICPR13, NICPR20, NICPR21, NICPR293 & NICPR294** | **0.97** |
| **6** | **594** | **GVSVITPGTNTSNQVA** | **WT SARS-CoV-2, NICPR13, NICPR20, NICPR21, NICPR293 & NICPR294** | **0.95** |
| **8** | **257** | **GWTAGAAAYYVGYLQP / SWTAGAAAYYVGYLQP (254)** | **Present in WT SARS-CoV-2, NICPR294, NICPR21/ WT SARS-CoV-2, NICPR13, NICPR20, NICPR21, NICPR293 & NICPR294** | **0.95** |
| **10** | **1112** | **PQIITTDNTFVSGNCD** | **WT SARS-CoV-2, NICPR13, NICPR20, NICPR21, NICPR293 & NICPR294** | **0.95** |
| **12** | **245** | **HRSYLTPGDSSSGWTA / HRSYLTPGDSSSSWTA** | **WT SARS-CoV-2, NICPR294, NICPR21/ WT SARS-CoV-2, NICPR13, NICPR20, NICPR21, NICPR293 & NICPR294** | **0.92** |
| **14** | **1180** | **QKEIDRLNEVAKNLNE** | **WT SARS-CoV-2, NICPR13, NICPR20, NICPR21, NICPR293 & NICPR294** | **0.92** |
| **16** | **476/470** | **GSTPCNGVEGFNCYFP/ TEIYQAGSTPCNGVEG** | **Only in WT SARS-CoV-2** | **0.91** |
| **18** | **307** | **TVEKGIYQTSNFRVQP** | **WT SARS-CoV-2, NICPR13, NICPR20, NICPR21, NICPR293 & NICPR294** | **0.91** |
| **20** | **648** | **GCLIGAEHVNNSYECD** | **WT SARS-CoV-2** | **0.9** |
| **22** | **492** | **LQSYGFQPTNGVGYQP** | **WT SARS-CoV-2 only** | **0.9** |
| **24** | **1247** | **CCSCGSCCKFDEDDSE** | **WT SARS-CoV-2, NICPR13, NICPR20, NICPR21, NICPR293 & NICPR294** | **0.9** |
| **26** | **470** | **TEIYQAGSTPCNGVEG** | **Only in WT SARS-CoV-2** | **0.89** |
| **28** | **236** | **TRFQTLLALHRSYLTP** | **WT SARS-CoV-2, NICPR13, NICPR20, NICPR21, NICPR293 & NICPR294** | **0.89** |
| **30** | **1206** | **YEQYIKWPWYIWLGFI /**  **YEQYIKWPWYIWLVFI (1201)** | **WT SARS-CoV-2, NICPR13, NICPR20, NICPR21, & NICPR294 / WT SARS-CoV-2, NICPR13, NICPR20, NICPR21, NICPR293 & NICPR294** | **0.89** |
| **32** | **1058** | **HGVVFLHVTYVPAQEK** | **WT SARS-CoV-2, NICPR13, NICPR20, NICPR21, NICPR293 & NICPR294** | **0.89** |
| **34** | **931** | **IGKIQDSLSSTASALG** | **WT SARS-CoV-2, NICPR13, NICPR20, NICPR21, NICPR293 & NICPR294** | **0.88** |
| **36** | **898** | **FAMQMAYRFNGIGVTQ** | **WT SARS-CoV-2, NICPR13, NICPR20, NICPR21, NICPR293 & NICPR294** | **0.88** |
| **38** | **1064** | **HVTYVPAQEKNFTTAP** | **WT SARS-CoV-2, NICPR13, NICPR20, NICPR21, NICPR293 & NICPR294** | **0.88** |
| **40** | **786** | **KQIYKTPPIKDFGGFN** | **Only in WT SARS-CoV-2** | **0.87** |
| **42** | **525** | **CGPKKSTNLVKNKCVN** | **WT SARS-CoV-2, NICPR13, NICPR20, NICPR21, NICPR293 & NICPR294** | **0.86** |
| **44** | **464** | **FERDISTEIYQAGSTP / FERDISTEIYQAGNKP** | **WT SARS-CoV-2 only** | **0.86** |
| **46** | **266** | **YVGYLQPRTFLLKYNE** | **WT SARS-CoV-2, NICPR13, NICPR20, NICPR21, NICPR293 & NICPR294** | **0.86** |
| **48** | **151** | **SWMESEFRVYSSANNC** | **WT SARS-CoV-2, NICPR294, NICPR21** | **0.86** |
| **50** | **1240** | **CCSCLKGCCSCGSCCK** | **WT SARS-CoV-2, NICPR13, NICPR20, NICPR21, NICPR293 & NICPR294** | **0.86** |
| **52** | **732** | **TKTSVDCTMYICGDST** | **WT SARS-CoV-2, NICPR13, NICPR20, NICPR21, NICPR293 & NICPR294** | **0.85** |
| **54** | **406** | **EVRQIAPGQTGKIADY / EVRQIAPGQTGNIADY (394)** | **WT SARS-CoV-2/ NICPR21** | **0.85** |
| **56** | **391** | **CFTNVYADSFVIRGDE / CFTNVYADSFVIRGNE** | **WT SARS-CoV-2, NICPR21 and / WT SARS-CoV-2, NICPR13, NICPR20, NICPR21, NICPR293 & NICPR294** | **0.85** |
| **58** | **1051** | **MSFPQSAPHGVVFLHV** | **WT SARS-CoV-2, NICPR13, NICPR20, NICPR21, NICPR293 & NICPR294** | **0.85** |
| **60** | **97** | **KSNIIRGWIFGTTLDS** | **WT SARS-CoV-2, NICPR13, NICPR20, NICPR21, NICPR293 & NICPR294** | **0.84** |
| **62** | **739** | **TMYICGDSTECSNLLL** | **WT SARS-CoV-2, NICPR13, NICPR20, NICPR21, NICPR293 & NICPR294** | **0.84** |
| **64** | **630** | **TPTWRVYSTGSNVFQT** | **WT SARS-CoV-2, NICPR13, NICPR20, NICPR21, NICPR293 & NICPR294** | **0.84** |
| **66** | **415** | **TGKIADYNYKLPDDFT** | **WT SARS-CoV-2 only** | **0.84** |
| **68** | **200** | **YFKIYSKHTPINLVRD/**  **YFKIYSKHTPINLGRD/**  **YFKIYSKHTPVNLGRD/**  **YFKIYSKHTPIIVREP/**  **FKNIDGYFKIYSKHTP** | **WT SARS-CoV-2 only/ NICPR294 (195)/ NICPR20 (197), NICPR293 (195)/ NICPR13 (186)/ WT SARS-CoV-2, NICPR13, NICPR20, NICPR21, NICPR293 & NICPR294** | **0.84** |
| **70** | **1234** | **LCCMTSCCSCLKGCCS** | **WT SARS-CoV-2, NICPR13, NICPR20, NICPR21, NICPR293 & NICPR294** | **0.84** |
| **72** | **1084** | **DGKAHFPREGVFVSNG** | **WT SARS-CoV-2, NICPR13, NICPR20, NICPR21, NICPR293 & NICPR294** | **0.84** |
| **74** | **847** | **RDLICAQKFNGLTVLP/**  **RDLICAQKFKGLTVLP (835)** | **WT SARS-CoV-2, NICPR20, NICPR21, NICPR293 & NICPR294/ NICPR13 (835)** | **0.83** |
| **76** | **604** | **TSNQVAVLYQDVNCTE** | **WT SARS-CoV-2 only** | **0.83** |
| **78** | **280** | **NENGTITDAVDCALDP** | **WT SARS-CoV-2, NICPR13, NICPR20, NICPR21, NICPR293 & NICPR294** | **0.83** |
| **80** | **1195** | **ESLIDLQELGKYEQYI** | **WT SARS-CoV-2, NICPR13, NICPR293 & NICPR294** | **0.83** |
| **82** | **1129** | **VIGIVNNTVYDPLQPE** | **WT SARS-CoV-2, NICPR13, NICPR20, NICPR21, NICPR293 & NICPR294** | **0.83** |
| **84** | **70** | **VSGTNGTKRFDNPVLP/**  **ISGTNGTKRFDNPVLP/**  **HXXSGTNGTKRFDNPV** | **WT SARS-CoV-2, NICPR20 & NICPR21/ NICPR13 (68) & NICPR293 (65)/ NICPR294 (63)** | **0.82** |
| **86** | **689** | **SQSIIAYTMSLGAENS** | **WT SARS-CoV-2, NICPR13, NICPR20, NICPR21, NICPR293 & NICPR294** | **0.82** |
| **88** | **564** | **QFGRDIADTTDAVRDP** | **WT SARS-CoV-2, NICPR13, NICPR20, NICPR21, NICPR293 & NICPR294** | **0.82** |
| **90** | **329** | **FPNITNLCPFGEVFNA/**  **FPNITNLCPFDEVFNA/**  **FPNITNLCPFHEVFNA** | **WT SARS-CoV-2/ NICPR294 (324), NICPR13 (317)/ NICPR21 (326), NICPR20 (326)** | **0.82** |
| **92** | **347** | **FASVYAWNRKRISNCV** | **WT SARS-CoV-2, NICPR13, NICPR20, NICPR21, NICPR293 & NICPR294** | **0.81** |
| **94** | **1041** | **DFCGKGYHLMSFPQSA** | **WT SARS-CoV-2, NICPR13, NICPR20, NICPR21, NICPR293 & NICPR294** | **0.81** |
| **96** | **803** | **SQILPDPSKPSKRSFI** | **WT SARS-CoV-2, NICPR13, NICPR20, NICPR21, NICPR293 & NICPR294** | **0.8** |
| **98** | **674** | **YQTQTNSPRRARSVAS/**  **IGAGICASYQTQTNSP (666)** | **WT SARS-CoV-2 only** | **0.8** |
| **100** | **583** | **EILDITPCSFGGVSVI** | **WT SARS-CoV-2, NICPR13, NICPR20, NICPR21, NICPR293 & NICPR294** | **0.8** |
| **102** | **49** | **HSTQDLFLPFFSNVTW** | **WT SARS-CoV-2, NICPR13, NICPR20, NICPR21, NICPR293 & NICPR294** | **0.8** |
| **104** | **320** | **VQPTESIVRFPNITNL** | **WT SARS-CoV-2, NICPR13, NICPR20, NICPR21, NICPR293 & NICPR294** | **0.8** |
| **106** | **288** | **AVDCALDPLSETKCTL** | **WT SARS-CoV-2, NICPR13, NICPR20, NICPR21, NICPR293 & NICPR294** | **0.8** |
| **108** | **194** | **FKNIDGYFKIYSKHTP** | **WT SARS-CoV-2, NICPR13, NICPR20, NICPR21, NICPR293 & NICPR294** | **0.8** |
| **110** | **124** | **TNVVIKVCEFQFCNDP** | **WT SARS-CoV-2, NICPR13, NICPR20, NICPR21, NICPR293 & NICPR294** | **0.8** |
| **112** | **829** | **ADAGFIKQYGDCLGDI** | **WT SARS-CoV-2, NICPR13, NICPR20, NICPR21, NICPR293 & NICPR294** | **0.79** |
| **114** | **360** | **NCVADYSVLYNSASFS/**  **NCVADYSVLYNFAPFF/**  **RKRISNCVADYSVLYN** | **WT SARS-CoV-2/ NICPR294 (355), NICPR21 (357), NICPR293 (355), NICPR20 (357)/ NICPR13 (343)** | **0.79** |
| **116** | **19** | **TTRTQLPPAYTNSFTR/**  **VSSQCVNLTTRTQLPP (11)** | **WT SARS-CoV-2 and NICPR13 only/ WT SARS-CoV-2 (11) and NICPR13 (11) only** | **0.79** |
| **118** | **159** | **VYSSANNCTFEYVSQP** | **WT SARS-CoV-2, NICPR20, NICPR21, NICPR293 & NICPR294** | **0.78** |
| **120** | **107** | **GTTLDSKTQSLLIVNN** | **WT SARS-CoV-2, NICPR13, NICPR20, NICPR21, NICPR293 & NICPR294** | **0.78** |
| **122** | **994** | **DRLITGRLQSLQTYVT** | **WT SARS-CoV-2, NICPR13, NICPR20, NICPR21, NICPR293 & NICPR294** | **0.77** |
| **124** | **719** | **TISVTTEILPVSMTKT** | **WT SARS-CoV-2, NICPR13, NICPR20, NICPR21, NICPR293 & NICPR294** | **0.77** |
| **126** | **657** | **NNSYECDIPIGAGICA** | **WT SARS-CoV-2, NICPR13, NICPR20, NICPR21, NICPR293 & NICPR294** | **0.77** |
| **128** | **501** | **NGVGYQPYRVVVLSFE/**  **YGVGYQPYRVVVLSFE (498)** | **WT SARS-CoV-2 only/ NICPR20 (498)** | **0.77** |
| **130** | **1227** | **IVMVTIMLCCMTSCCS** | **WT SARS-CoV-2, NICPR13, NICPR20, NICPR21, NICPR293 & NICPR294** | **0.77** |
| **132** | **941** | **TASALGKLQDVVNQNA/**  **TASALGKLQDVVNHNA** | **WT SARS-CoV-2/ NICR294, NICPR13, NICPR21, NICPR293, NICPR20.** | **0.76** |
| **134** | **838** | **GDCLGDIAARDLICAQ** | **WT SARS-CoV-2, NICPR13, NICPR20, NICPR21, & NICPR294** | **0.76** |
| **136** | **709** | **NNSIAIPTNFTISVTT** | **WT SARS-CoV-2, NICPR13, NICPR20, NICPR21, NICPR293 & NICPR294** | **0.76** |
| **138** | **695** | **YTMSLGAENSVAYSNN** | **WT SARS-CoV-2, NICPR13, NICPR20, NICPR21, NICPR293 & NICPR294** | **0.76** |
| **140** | **374** | **FSTFKCYGVSPTKLND** | **WT SARS-CoV-2 only** | **0.76** |
| **142** | **301** | **CTLKSFTVEKGIYQTS** | **WT SARS-CoV-2, NICPR13, NICPR20, NICPR21, NICPR293 & NICPR294** | **0.76** |
| **144** | **251** | **PGDSSSGWTAGAAAYY** | **WT SARS-CoV-2, NICPR294 & NICPR13 only.** | **0.76** |
| **146** | **1146** | **DSFKEELDKYFKNHTS** | **WT SARS-CoV-2, NICPR13, NICPR20, NICPR21, NICPR293 & NICPR294** | **0.76** |
| **148** | **778** | **TQEVFAQVKQIYKTPP** | **WT SARS-CoV-2, NICPR13, NICPR20, NICPR21, NICPR293 & NICPR294** | **0.75** |
| **150** | **666** | **IGAGICASYQTQTNSP** | **WT SARS-CoV-2 only** | **0.75** |
| **152** | **1139** | **DPLQPELDSFKEELDK** | **WT SARS-CoV-2, NICPR13, NICPR20, NICPR21, NICPR293 & NICPR294** | **0.75** |
| **154** | **815** | **RSFIEDLLFNKVTLAD** | **WT SARS-CoV-2, NICPR13, NICPR20, NICPR21, NICPR293 & NICPR294** | **0.74** |
| **156** | **765** | **RALTGIAVEQDKNTQE** | **WT SARS-CoV-2, NICPR13, NICPR20, NICPR21, NICPR293 & NICPR294** | **0.74** |
| **158** | **366** | **SVLYNSASFSTFKCYG** | **WT SARS-CoV-2 only** | **0.74** |
| **160** | **34** | **RGVYYPDKVFRSSVLH** | **WT SARS-CoV-2, NICPR13, NICPR20, NICPR21, NICPR293 & NICPR294** | **0.74** |
| **162** | **1103** | **FVTQRNFYEPQIITTD** | **WT SARS-CoV-2, NICPR13, NICPR20, NICPR21, NICPR293 & NICPR294** | **0.74** |
| **164** | **988** | **EAEVQIDRLITGRLQS** | **WT SARS-CoV-2, NICPR13, NICPR20, NICPR21, NICPR293 & NICPR294** | **0.73** |
| **166** | **860** | **VLPPLLTDEMIAQYTS** | **WT SARS-CoV-2, NICPR13, NICPR20, NICPR21, NICPR293 & NICPR294** | **0.73** |
| **168** | **547** | **TGTGVLTESNKKFLPF/**  **NGLKGTGVLTESNKKF (s13/532)** | **WT SARS-CoV-2, NICPR13, NICPR20, NICPR21, NICPR293 & NICPR294/ NICPR13 (532)** | **0.73** |
| **170** | **484** | **EGFNCYFPLQSYGFQP/**  **AGFNCYFPLQSYGFRP (479)** | **WT SARS-CoV-2 and / NICPR293 (479)** | **0.73** |
| **172** | **136** | **CNDPFLGVYYHKNNKS** | **WT SARS-CoV-2 only** | **0.73** |
| **174** | **1166** | **LGDISGINASVVNIQK** | **WT SARS-CoV-2, NICPR13, NICPR20, NICPR21, NICPR293 & NICPR294** | **0.73** |
| **176** | **1009** | **TQQLIRAAEIRASANL** | **WT SARS-CoV-2, NICPR13, NICPR20, NICPR21, NICPR293 & NICPR294** | **0.73** |
| **178** | **88** | **DGVYFASTEKSNIIRG** | **WT SARS-CoV-2, NICPR13, NICPR20, NICPR21, NICPR293 & NICPR294** | **0.72** |
| **180** | **747** | **TECSNLLLQYGSFCTQ** | **WT SARS-CoV-2, NICPR13, NICPR20, NICPR21, NICPR293 & NICPR294** | **0.72** |
| **182** | **1094** | **VFVSNGTHWFVTQRNF** | **WT SARS-CoV-2, NICPR13, NICPR20, NICPR21, NICPR293 & NICPR294** | **0.72** |
| **184** | **953** | **NQNAQALNTLVKQLSS/**  **NHNAQALNTLVKQLSS** | **WT SARS-CoV-2/ NICPR294, NICPR13, NICPR21, NICPR293 & NICPR20** | **0.71** |
| **186** | **636** | **YSTGSNVFQTRAGCLI** | **WT SARS-CoV-2, NICPR13, NICPR20, NICPR21, NICPR293 & NICPR294** | **0.71** |
| **188** | **445** | **VGGNYNYLYRLFRKSN/**  **KVSGNYNYLYRLFRKS (432)** | **WT SARS-CoV-2/ NICPR13(432), NICPR21(441), NICPR293(439) & NICPR20(441).** | **0.71** |
| **190** | **430** | **TGCVIAWNSNNLDSKV/**  **TGCVIAWNSNKLDSKV** | **WT SARS-CoV-2/ NICPR13 (418), NICPR21 (427), NICPR20 (427), NICPR293 (425), NICPR294(425).** | **0.71** |
| **192** | **423** | **YKLPDDFTGCVIAWNS** | **WT SARS-CoV-2 only** | **0.71** |
| **194** | **1001** | **LQSLQTYVTQQLIRAA** | **WT SARS-CoV-2, NICPR13, NICPR20, NICPR21, NICPR293 & NICPR294** | **0.71** |
| **196** | **612** | **YQDVNCTEVPVAIHAD** | **WT SARS-CoV-2 only** | **0.7** |
| **198** | **25** | **PPAYTNSFTRGVYYPD/**  **TRTQSYTNSFTRGVYY** | **WT SARS-CoV-2 & NICPR13/ NICPR294 (20), NICPR21 (20), NICPR20 (20), NICPR293 (20)** | **0.7** |
| **200** | **1201** | **PWYIWLGFIAGLIAIV** | **WT SARS-CoV-2, NICPR13, NICPR20, NICPR21, & NICPR294** | **0.7** |
| **202** | **1121** | **FVSGNCDVVIGIVNNT** | **WT SARS-CoV-2, NICPR13, NICPR20, NICPR21, NICPR293 & NICPR294** | **0.7** |
| **204** | **890** | **AGAALQIPFAMQMAYR** | **WT SARS-CoV-2, NICPR13, NICPR20, NICPR21, NICPR293 & NICPR294** | **0.69** |
| **206** | **400** | **FVIRGDEVRQIAPGQT** | **WT SARS-CoV-2 Only** | **0.69** |
| **208** | **1153** | **DKYFKNHTSPDVDLGD** | **WT SARS-CoV-2, NICPR13, NICPR20, NICPR21, NICPR293 & NICPR294** | **0.69** |
| **210** | **1078** | **APAICHDGKAHFPREG** | **WT SARS-CoV-2, NICPR13, NICPR20, NICPR21, NICPR293 & NICPR294** | **0.69** |
| **212** | **294** | **DPLSETKCTLKSFTVE** | **WT SARS-CoV-2, NICPR13, NICPR20, NICPR21, NICPR293 & NICPR294** | **0.68** |
| **214** | **573** | **TDAVRDPQTLEILDIT** | **WT SARS-CoV-2, NICPR13, NICPR20, NICPR21, NICPR293 & NICPR294** | **0.67** |
| **216** | **910** | **GVTQNVLYENQKLIAN** | **WT SARS-CoV-2, NICPR13, NICPR20, NICPR21, NICPR293 & NICPR294** | **0.66** |
| **218** | **771** | **AVEQDKNTQEVFAQVK** | **WT SARS-CoV-2, NICPR13, NICPR20, NICPR21, NICPR293 & NICPR294** | **0.66** |
| **220** | **511** | **VVLSFELLHAPATVCG** | **WT SARS-CoV-2, NICPR13, NICPR20, NICPR21, NICPR293 & NICPR294** | **0.66** |
| **222** | **3** | **VFLVLLPLVSSQCVNL** | **WT SARS-CoV-2, NICPR13, NICPR20, NICPR21, NICPR293 & NICPR294** | **0.66** |
| **224** | **1159** | **HTSPDVDLGDISGINA** | **WT SARS-CoV-2, NICPR13, NICPR20, NICPR21, NICPR293 & NICPR294** | **0.66** |
| **226** | **868** | **EMIAQYTSALLAGTIT** | **WT SARS-CoV-2, NICPR13, NICPR20, NICPR21, NICPR293 & NICPR294** | **0.65** |
| **228** | **457** | **RKSNLKPFERDISTEI** | **WT SARS-CoV-2, NICPR294 & NICPR13 only** | **0.65** |
| **230** | **224** | **EPLVDLPIGINITRFQ** | **WT SARS-CoV-2, NICPR13, NICPR20, NICPR21, NICPR293 & NICPR294** | **0.65** |
| **232** | **174** | **PFLMDLEGKQGNFKNL/**  **NCLMDLEGKQGNFKNL (160)** | **WT SARS-CoV-2, NICPR20, NICPR21, NICPR293 & NICPR294/ NICPR13 (160)** | **0.65** |
| **234** | **11** | **VSSQCVNLTTRTQLPP** | **WT SARS-CoV-2 and NICPR13 only** | **0.65** |
| **236** | **975** | **SVLNDILSRLDKVEAE** | **WT SARS-CoV-2, NICPR20, NICPR21, NICPR293 & NICPR294** | **0.64** |
| **238** | **1257** | **DEDDSEPVLKGVKLHY** | **WT SARS-CoV-2, NICPR13, NICPR20, NICPR21, NICPR293 & NICPR294** | **0.64** |
| **240** | **964** | **KQLSSNFGAISSVLND** | **WT SARS-CoV-2 only** | **0.63** |
| **242** | **82** | **PVLPFNDGVYFASTEK** | **WT SARS-CoV-2, NICPR13, NICPR20, NICPR21, NICPR293 & NICPR294** | **0.63** |
| **244** | **76** | **TKRFDNPVLPFNDGVY** | **WT SARS-CoV-2, NICPR13, NICPR20, NICPR21, NICPR293 & NICPR294** | **0.63** |
| **246** | **384** | **PTKLNDLCFTNVYADS** | **WT SARS-CoV-2, NICPR13, NICPR20, NICPR21, NICPR293 & NICPR294** | **0.62** |
| **248** | **43** | **FRSSVLHSTQDLFLPF** | **WT SARS-CoV-2, NICPR13, NICPR20, NICPR21, NICPR293 & NICPR294** | **0.61** |
| **250** | **1032** | **CVLGQSKRVDFCGKGY** | **WT SARS-CoV-2, NICPR13, NICPR20, NICPR21, NICPR293 & NICPR294** | **0.61** |
| **252** | **1026** | **ATKMSECVLGQSKRVD** | **WT SARS-CoV-2, NICPR13, NICPR20, NICPR21, NICPR293 & NICPR294** | **0.61** |
| **254** | **1020** | **ASANLAATKMSECVLG** | **WT SARS-CoV-2, NICPR13, NICPR20, NICPR21, NICPR293 & NICPR294** | **0.6** |
| **256** | **210** | **INLVRDLPQGFSALEP** | **WT SARS-CoV-2 only** | **0.59** |
| **258** | **757** | **GSFCTQLNRALTGIAV** | **WT SARS-CoV-2 only** | **0.57** |
| **260** | **182** | **KQGNFKNLREFVFKNI** | **WT SARS-CoV-2, NICPR13, NICPR20, NICPR21, NICPR293 & NICPR294** | **0.57** |
| **262** | **118** | **LIVNNATNVVIKVCEF** | **WT SARS-CoV-2, NICPR13, NICPR20, NICPR21, NICPR293 & NICPR294** | **0.55** |
| **264** | **922** | **LIANQFNSAIGKIQDS** | **WT SARS-CoV-2, NICPR13, NICPR20, NICPR21, NICPR293 & NICPR294** | **0.54** |
| **266** | **792** | **PPIKDFGGFNFSQILP** | **WT SARS-CoV-2 only** | **0.52** |
